# Supplementary material for: Effectiveness of pneumococcal and influenza vaccines to prevent serious health complications in adults with chronic liver disease: a protocol for a systematic review
Source: BMJ Open. 2018 Mar 16;8(3):e018223. doi: 10.1136/bmjopen-2017-018223 (PMC5857657; doi:10.1136/bmjopen-2017-018223)
Supplement: Author's manuscript [file bmjopen-2017-018223.draft_revisions.pdf]

BMJ Open is committed to open peer review. As part of this commitment we make the peer review history of every article we publish publicly available.

When an article is published we post the peer reviewers' comments and the authors' responses online. We also post the versions of the paper that were used during peer review. These are the versions that the peer review comments apply to.

The versions of the paper that follow are the versions that were submitted during the peer review process. They are not the versions of record or the final published versions. They should not be cited or distributed as the published version of this manuscript.

BMJ Open is an open access journal and the full, final, typeset and author-corrected version of record of the manuscript is available on our site with no access controls, subscription charges or pay-per-view fees (http://bmjopen.bmj.com).

If you have any questions on BMJ Open's open peer review process please email info.bmjopen@bmj.com

BMJ Open

EFFECTIVENESS OF PNEUMOCOCCAL AND INFLUENZA VACCINES TO PREVENT SERIOUS HEALTH COMPLICATIONS IN ADULTS WITH CHRONIC LIVER DISEASE: A PROTOCOL FOR A SYSTEMATIC REVIEW

Journal:	*BMJ Open*
Manuscript ID	bmjopen-2017-018223
Article Type:	Protocol
Date Submitted by the Author:	14-Jun-2017
Complete List of Authors:	Härmälä, Suvi; University College London, Institute of Health Informatics<br>Parisinos, Constantinos; University College London, Institute of Health Informatics<br>Shallcross, Laura; University College London, Institute of Health Informatics<br>O'Brien, Alastair; University College London, Division of Medicine<br>Hayward, Andrew; University College London, Institute of Health Informatics; University College London, Institute of Epidemiology and Health Care
**Primary Subject Heading**:	Infectious diseases
Secondary Subject Heading:	Gastroenterology and hepatology
Keywords:	Pneumococcal vaccine, Influenza vaccine, Chronic liver disease

SCHOLARONE™
Manuscripts

For peer review only - http://bmjopen.bmj.com/site/about/guidelines.xhtml

EFFECTIVENESS OF PNEUMOCOCCAL AND INFLUENZA VACCINES TO PREVENT SERIOUS HEALTH COMPLICATIONS IN ADULTS WITH CHRONIC LIVER DISEASE: A PROTOCOL FOR A SYSTEMATIC REVIEW

Suvi Härmälä[1], Constantinos Parisinos[1], Laura Shallcross[1], Alastair O'Brien[2] & Andrew Hayward[1,3]

[1] Institute of Health Informatics, University College London, London, United Kingdom.

[2] Division of Medicine, University College London, London, United Kingdom.

[3] Institute of Epidemiology and Health Care, University College London, London, United Kingdom.

Corresponding author: Suvi K. Härmälä, Institute of Health Informatics, University College London, 222 Euston Road, London NW1 2DA, United Kingdom, suvi.harmala.14@ucl.ac.uk, +44 (0)20 3549 5338

Keywords: Pneumococcal vaccine, influenza vaccine, chronic liver disease

Word count (excluding title page, abstract, strengths & limitations, references and table): 3909

For peer review only

ABSTRACT

Introduction: In advanced chronic liver disease, diseases caused by common bacteria *Streptococcus pneumoniae* or influenza virus put people at an increased risk of serious health complications and death. The effectiveness of the available vaccines in reducing the risk of poor health outcomes, however, is less clear.

Methods and analysis: We will search MEDLINE, EMBASE, Pubmed, Cochrane Central Register of Controlled Trials (CENTRAL), The Cochrane Hepato-Biliary Group Specialised Register and The Cochrane Acute Respiratory Infections Group Register of Trials for published reports on randomised controlled trials and observational studies on the effectiveness of pneumococcal and influenza vaccines in people with chronic liver disease. Two independent reviewers will screen the studies for eligibility, extract data and assess study quality and risk of bias. Random effects meta-analyses will be performed as appropriate.

Ethics and dissemination: Formal ethical approval is not required, as no primary data will be collected for this study. We will publish results of this study in relevant peer-reviewed medical journal or journals. Where possible, the study results will also be presented as posters or talks at relevant medical conferences and meetings.

Prospero registration number: CRD42017067277

For peer review only

STRENGTHS AND LIMITATIONS OF THIS STUDY:

- This study protocol follows the recommendations by the Preferred Reporting Items for Systematic Review and Meta-Analysis Protocols (PRISMA-P).
- This study protocol has been prospectively registered in the International Prospective Register of Systematic Reviews (PROSPERO).
- Our comprehensive search strategy will minimise the risk of missing relevant studies, in particular, those with a randomised design.
- The selection of studies, data extraction, the risk of bias and quality of evidence assessments will be conducted by two independent authors.
- Inclusion of studies with a non-randomised design may decrease the overall quality of the body of evidence for the study outcomes.

For peer review only

BACKGROUND

Burden of pneumococcal and influenza infections in chronic liver disease

In advanced chronic liver disease, as the immune function progressively deteriorates, diseases caused by common bacteria *Streptococcus pneumoniae* or influenza virus can lead to serious health complications and death.

In Spain in 2011, the population-level annual incidence rate for pneumococcal pneumonia-related hospitalisation in adults with liver disease was estimated at approximately 540 per 100 000 compared to approximately 6 per 100 000 without at-risk conditions,[1]. Adults with liver disease were over 50 times more likely to be hospitalised for pneumococcal pneumonia than adults without at-risk conditions,[1]. Similarly, in England in 2008/2009, for invasive pneumococcal disease (IPD), such as meningitis, bacteraemia and sepsis, the annual incidence of hospitalisation in adults with liver disease was estimated at about 100 per 100 000 compared to about 8 per 100 000 in the healthy population,[2]. Approximately 37% of liver disease patients hospitalised for IPD died, compared to 5% of patients without underlying risk conditions,[2]. Chronic liver disease patients were over 30 times more likely to be admitted to hospital and 10 times more likely to die during the IPD-related hospitalisation than adults without at-risk conditions,[2].

Although there are no population-level estimates of severe influenza incidence in people with chronic liver disease, evidence suggests that liver disease patients are at an increased risk of health complications from influenza. A 2-fold increased risk of influenza admission was observed in liver disease patients at 19 hospitals in Russia, Turkey, China, and Spain during the 2013/2014 season,[3]. Similarly, an analysis of data on laboratory-confirmed influenza cases collected from several World Health Organisation (WHO) member states during the 2009 influenza A (H1N1) pandemic found liver disease patients to have a greater than 5-fold increased risk of influenza-related hospitalisation and over 17-fold increased risk of death compared to that of healthy individuals,[4].

For peer review only - http://bmjopen.bmj.com/site/about/guidelines.xhtml

Furthermore, influenza infection, while not directly targeting the liver, may cause collateral transient liver damage,[5] and trigger hepatic decompensation (such as ascites and hepatic encephalopathy) in liver disease patients,[6].

Types of pneumococcal and influenza vaccines, vaccination policy and vaccine uptake

Two types of vaccines, polysaccharide pneumococcal vaccines including serotypes of *S. pneumoniae* (PPV23) and conjugate pneumococcal vaccines including 7 (PCV7), 10 (PCV10) or 13 (PCV13) *S. pneumoniae* serotypes, are available to protect against pneumococcal infection. None of these contains live bacteria.

The most commonly used influenza vaccines are injectable, inactivated vaccines that contain either inactivated whole influenza virus or split or subunit virus products. These vaccines protect either against two influenza A (H1N1 and H3N2) strains and one influenza B (Victoria and Yamagata lineages) strain (trivalent vaccines) or two influenza A and two influenza B strains (quadrivalent vaccines). New vaccines are developed every year to protect against the prevailing strains of the upcoming influenza season and a yearly vaccination is recommended to ensure continued protection. Live attenuated influenza vaccines exist, however, these may not be suitable for people with chronic co-morbidities,[7].

The majority of European countries recommend both adult influenza (29/29 countries) and pneumococcal vaccination (22/29 countries) for specific high-risk groups,[8]. Chronic liver disease patients, however, may not be included in these high-risk target groups in all countries. Whilst 90% (27/ 30) of the countries recommended influenza immunisation for people with liver disease during the 2014-2015 influenza season,[9], only 43% (6/14) of countries surveyed in 2005 recommended pneumococcal vaccination for chronic liver disease patients,[10]. Moreover, whilst the adult vaccination recommendations for influenza are supported by official funding mechanisms in most countries

For peer review only - http://bmjopen.bmj.com/site/about/guidelines.xhtml

(21/29), the cost of the pneumococcal vaccination is covered by only half (11/22),[8].

Uptake of influenza vaccine in people with chronic diseases in general is poor. The median coverage rate across Europe in the 2014/2015 season was less than 50% (only 7/30 countries were able to provide separate coverage data for individuals with chronic medical conditions),[9]. The situation in liver disease patients as a separate group seems no different with less than 50% of working-age liver disease patients in the UK covered by the influenza vaccine in 2015/2016 season,[11]. Although fewer data exist concerning the uptake of pneumococcal adult vaccination,[8], its uptake in liver disease patients is not likely to be any higher than that of influenza vaccine.

Rationale for the review

Approximately 29 million people in Europe alone are affected by some form of liver disease,[12]. Worryingly, due to the increasing prevalence of obesity and persistence of other liver damage risk factors such as alcohol abuse and hepatitis infections, this number is expected to grow,[12]. Whilst evidence suggests that following infection with influenza or *S. pneumoniae*, people with liver disease have a higher than average risk of poor health outcomes, the effectiveness of the vaccines in reducing this risk is less clear and warrants further investigation.

To our knowledge, no systematic review to date has investigated the effects of pneumococcal and influenza vaccines in preventing poor health outcomes in chronic liver disease. The present review intends to fill this gap by providing a systematic synthesis of the available evidence. The results of this review may inform future vaccination strategies and help improve vaccination coverage. This, in turn, may have a positive impact on both the number of influenza and pneumococcal disease-related hospital admissions and patient outcomes in liver disease.

For peer review only - http://bmjopen.bmj.com/site/about/guidelines.xhtml

OBJECTIVES

The aim of this review is to assess the effectiveness of pneumococcal and influenza vaccines to prevent serious health complications in adults with chronic liver disease.

The objectives are:

- To assess the effectiveness of pneumococcal and influenza vaccines to prevent hospitalisation in adults with chronic liver disease.
- To assess the effectiveness of pneumococcal and influenza vaccines to prevent death in adults with chronic liver disease.
- To assess the effects of pneumococcal and influenza vaccines for eliciting a serological response in adults with chronic liver disease.

METHODS

This study protocol follows the recommendations by the Preferred Reporting Items for Systematic Review and Meta-Analysis Protocols (PRISMA-P) 2015,[13].

Eligibility criteria

Types of studies

We will include all randomized clinical trials, cohort (with comparison group/s) and case-control studies that report the effects of pneumococcal or influenza vaccines for preventing infection or liver disease-related complications and death in adults with chronic liver disease. We will also include all randomized clinical trials, cohort (with or without comparison group/s) and case-control studies that report the serological response to one or both of these vaccines in adults with chronic liver disease. We will only include published studies in English language and studies that have been published or accepted for publication. We will exclude review articles, case reports, cross-sectional studies,

For peer review only - http://bmjopen.bmj.com/site/about/guidelines.xhtml

animal studies, editorials, clinical guidelines and any studies that have been fully or partially retracted from publication. Patients included in multiple studies will be reported only once.

Types of participants

We will include studies that enrol 18+ year-old adult patients with chronic liver disease of any severity (non-cirrhotic, cirrhotic) or aetiology (viral, alcoholic, non-alcoholic fatty liver, autoimmune).

Types of interventions

We will include studies that investigate the effects of a conjugate or polysaccharide pneumococcal vaccine (against *S. pneumoniae*) and/or an inactivated (whole virus, split virus or subunit), injectable influenza vaccine.

Types of comparators

We will include studies comparing one or both of the vaccines of interest to a placebo, an alternative intervention or no intervention. We will also include studies without a comparison group when the outcome studied is the serological response to the vaccine.

Types of outcome measures

We will include studies that report on one or more of our primary outcomes and/or our secondary outcome of interest.

Primary outcomes

- All-cause hospitalisation
- All-cause mortality

Secondary outcomes

For peer review only - http://bmjopen.bmj.com/site/about/guidelines.xhtml

- Serological response to vaccine
- Acute respiratory illness-related hospitalisation
- Influenza illness or influenza-like-illness (ILI)-related hospitalisation
- Pneumococcal disease-related hospitalisation
- Hospitalisation for liver disease complications (variceal bleeding, hepatic encephalopathy, ascites, spontaneous bacterial peritonitis, jaundice and bacteraemia or sepsis)
- Acute respiratory illness-related mortality
- Influenza illness or ILI-related mortality
- Pneumococcal disease-related mortality
- Liver disease-related mortality

Information sources

Electronic searches

To capture all relevant studies, we plan to search the following databases:

- Cochrane Central Register of Controlled Trials (CENTRAL)
- MEDLINE
- EMBASE
- PubMed
- The Cochrane Hepato-Biliary Group Specialised Register
- The Cochrane Acute Respiratory Infections Group Register of Trials

Each database will be searched separately and the search strategy first developed in MEDLINE will be adapted to each database interface as appropriate. We plan to also search relevant studies from the reference lists of the eligible studies identified through the electronic searches.

Search strategy

For peer review only

We will use two complementary strategies to identify relevant articles. First, we will identify articles reporting outcomes of vaccination in patients with liver disease by combining search terms for influenza and pneumococcal vaccination with search terms for chronic liver disease (Search 1). This search will be filtered by study design. The provisional search terms for liver disease and pneumococcal and influenza vaccines are listed in Table 1.
Recognising that liver disease patients may be included as subgroups in clinical trials of vaccination, we will also search for randomised controlled trials of influenza and pneumococcal vaccine that have recruited individuals from the general population (Search 2).

To search for studies with adult participants, we will combine the geriatric and adult medicine-specific search strategies by Kastner et al.,[14]. In order to maximise the sensitivity of the searches for randomised clinical trials, we will use a filter that combines terms from the Cochrane Highly Sensitive Search Strategy,[15] and less specific version of the same filter by Chalmers et al.,[16] to identify randomised trials. Similarly, to maximise the sensitivity of searches for case-control and cohort studies, we will use a filter that combines terms from the University of Texas School of Public Health filter for observational studies,[17], SIGN observational study filter,[18] and the BMJ Evidence Centre case-control and cohort strategy,[19]. We will limit our search to studies including adult human study subjects but not based on the setting/country or publication year of the articles. The search terms for adult and study design filters are listed in Table 1.

Table 1. MEDLINE (Ovid) provisional search terms

Search concept	Search terms
Pneumococcal vaccine	1. exp Pneumococcal Vaccines/
	2. exp Pneumococcal Infections/pc [Prevention & Control]
	3. ((anti?pneum* or pneum*) adj5 (vaccin* or immuni*)).mp.
	4. (PPV?23* or PPSV or PPSV?23* or PCV?7* or PCV?10* or

For peer review only

	PCV?13*).mp.
	5. ((PPV or PCV) adj5 (pneum* or vaccin* or immuni*)).mp.
	6. ((7?valent or hepta?valent or 10?valent or 13?valent or 23?valent) adj5 (vaccin* or immuni*)).mp.
	7. 1 or 2 or 3 or 4 or 5 or 6 or 7
Influenza vaccine	1. Influenza Vaccines/
	2. Influenza, Human/pc [Prevention & Control]
	3. ((anti?influenza or influenza or seasonal or anti?flu or flu) adj5 (vaccin* or immuni*)).mp.
	4. ((TIV or QIV or trivalent or quadrivalent or 3?valent or 4?valent) adj5 (vaccin* or immuni*)).mp.
	5. 1 or 2 or 3 or 4 or 5
Liver disease	1. exp Liver Diseases/
	2. ((liver or hepat*) adj3 disease*).mp.
	3. ("chronic liver" or "chronic hepat*").mp.
	4. cirrho*.mp.
	5. 1 or 2 or 3 or 4
Adult participants	1. exp Adult/
	2. adult.mp.
	3. (middle?aged or aged).sh.
	4. age*.tw.
	5. 1 or 2 or 3 or 4
Randomised controlled trials	1. randomized controlled trial.pt.
	2. randomi*.ab,ti.
	3. randomly.ab,ti.
	4. controlled clinical trial.pt.
	5. trial.ab,ti.
	6. groups.ab,ti.
	7. drug therapy.fs.
	8. placebo.ab,ti.

For peer review only

	9. 1 or 2 or3 or 4 or 5 or 6 or 7 or 8
	10. Animals/
	11. Humans/
	12. 10 not (10 and 11)
	13. 9 not 12
Case-control and cohort studies	1. Epidemiologic Studies/
	2. exp Case control studies/
	3. exp Cohort studies/
	4. Longitudinal studies/
	5. Follow up studies/
	6. Prospective studies/
	7. Retrospective studies/
	8. Control groups/
	9. Matched-Pair Analysis/
	10. (Case* adj5 control*).ti,ab,kw.
	11. (Case* adj5 comparison*).ti,ab,kw.
	12. Control group*.ti,ab,kw.
	13. (Cohort adj (study or studies)).ti,ab.
	14. Cohort anal*.ti,ab.
	15. (Follow up adj (study or studies)).ti,ab.
	16. (Observational adj (study or studies)).ti,ab.
	17. Longitudinal.ti,ab.
	18. Retrospective.ti,ab.
	19. Prospective.ti,ab.
	20. 1 or 2 or 3 or 4 or 5 or 6 or 7 or 8 or 9 or 10 or 11 or 12 or 13 or 14 or 15 or 16 or 17 or 18 or 19
	21. Animals/
	22. Humans/
	23. 21 not (21 and 22)
	24. 20 not 23

For peer review only

Study records

Data management

The search results will be uploaded into reference management software (Mendeley) to remove duplicate records of the same report. The unique records will then be uploaded into web-based, systematic review management software (DistillerSR). Both the initial abstract and title screening and the full-text review and extraction of data from the eligible studies will be performed using standardised, pre-created online forms. All forms will be piloted and revised as needed by the reviewers before starting the review.

Selection process

Articles that have been identified through the broad search of RCTs of pneumococcal or flu vaccination in the general population (Search 2) will first be pre-screened by title by one reviewer (SH). Articles meeting pre-screening criteria will be combined with the articles identified through Search 1. These articles will then all be screened by two independent reviewers (SH&CP) by abstract and title. Where the study eligibility cannot be established based on the title and abstract, the report will be passed on to the full-text review. Similarly, records subject to disagreement over eligibility will be included in the full-text review.

The full-text review will be independently completed for all eligible articles by two independent reviewers authors (SH&CP). Reasons for exclusion of ineligible studies will be recorded. Disagreements will be resolved by consulting a third review author (AO) and any uncertainties by correspondence with study investigators. Multiple reports of the same study will be collated into one and, where not possible, only the most relevant report based on our eligibility criteria will be included. The study selection process will be recorded and presented in flow diagram format according to the recommendations of Preferred Reporting Items for Systematic Reviews and Meta-Analyses (PRISMA),[20].

For peer review only

Data collection process

The data will be extracted and entered into standardised, pre-created online data extraction forms independently and in duplicate by two review authors (SH&CP). Disagreement will be resolved by consulting a third review author (LS) and uncertainties by correspondence with study investigators.

Data items

We will extract data on:

- Study participants: inclusion and exclusion criteria, method of recruitment/selection, study population characteristics and any imbalances at baseline (sex, age, aetiology and severity of liver disease, co-morbidities, alcohol use, smoking status, pre-vaccination infection status, medication/treatment other than intervention).
- Interventions and comparators (vaccine type, comparison treatment, dose, route of delivery, number and timing of vaccinations/comparator treatments, number of individuals in intervention and comparison group, follow-up time in intervention and comparison groups)
- Outcomes (definition, time points measured and reported, unit of measurement, number of outcomes in the intervention and control group, unadjusted and adjusted effect measures, covariates that the effect measures were adjusted for, comparisons, missing data and reasons for missingness, statistical methods used, processes for randomisation e.g. allocation concealment)
- Study designs and methods (study type, country and setting, date of study, study duration, aim of study, withdrawals).
- Study quality and study bias (according to the needs of the assessments specified below).
- Study funding and conflicts of interest

For peer review only - http://bmjopen.bmj.com/site/about/guidelines.xhtml

Effect measures will be collected in the format in which they are reported and transformed for presentation and analysis if appropriate.

Outcomes and prioritisation

Our main outcomes of interest are hospitalisation and death. These are potential severe outcomes of influenza illness and pneumococcal disease. It may be challenging to identify and establish, especially if hospital discharge records are reviewed retrospectively, the exact cause of the hospitalisation or death of a patient with an underlying chronic condition. For this reason, our primary outcomes we will include all causes. Additionally, studies may not always specify whether a patient with a diagnosis of an infectious disease or liver disease complication was actually hospitalised. We will assume that when a patient during the follow-up after the vaccination developed any liver disease complication (variceal bleeding, hepatic encephalopathy, ascites, jaundice, spontaneous bacterial peritonitis or bacteraemia or sepsis) or was diagnosed with IPD, they also required hospitalisation. Acute respiratory illness, non-invasive pneumococcal disease and influenza illness or ILI may not require hospitalisation so unless it is specified that the patient was hospitalised or the illness was recorded in the hospital records, we will assume the patient was not hospitalised.

Our secondary outcomes of interest are the serological response to pneumococcal and influenza vaccines and a range of cause-specific hospitalisation and mortality. Serological response to a vaccine is an indicator of the vaccine's effect on building protective immunity against the disease the vaccination targets. Since, however, it is difficult to know what level of antibody or increase in antibody concentration may provide protection in people with chronic liver disease, we will evaluate both the post-vaccination antibody level and the pre- to post-vaccination fold change in geometric mean antibody concentrations. This effect will be evaluated both for short-term (<6 months) and long-term (≥6 months) after vaccination. In case antibody responses are reported at multiple short-term or long-term time points, we will consider the

time point closest to the timing of vaccination as the as the short-term response and the time point closest to 6 months as the long-term response. The more detailed causes of hospitalisation and death, allow us to understand about the more specific effects of the vaccines.

Assessment of risk of bias in individual studies

We will use the Cochrane Collaborations tool,[15] for assessing the risk of bias in all studies included in the review after the full-text review. The level of risk of bias in random sequence generation, allocation concealment, blinding of participants and personnel, blinding of outcome assessment, incomplete outcome data, selective reporting and other sources will be judged as "low", "high" or "unclear" according to the criteria specified in the Cochrane Handbook,[15].

In the non-randomised studies, we will additionally assess the level of risk of confounding bias due to inadequately addressed differences between groups (i.e. is the effect estimate likely to be biased due to unaccounted confounding). We will consider age, sex, severity and aetiology of liver disease to be the most important potential confounders. We will judge the risk of confounding bias to be low if the study addressed the presence of these confounders by restricting participant selection by confounders, demonstrating balance between groups, matching on the confounders or adjusting for the confounders in statistical analyses of the effect size. The risk of confounding bias will be judged low if the confounders were adjusted for, high if the presence of the confounders was not addressed and unclear if there was insufficient information to judge the level of risk.

Two review authors (SH&CP) will independently assess the studies for each of the risk areas by entering a quote from the study to describe the procedures, their judgement together with a justification of the judgement into pre-created online forms in DistillerSR. Disagreements will be resolved by consulting a third review author (AH). The risk of bias assessments will be presented in a figure

For peer review only

that shows the level of risk in the different risk areas within each individual study and in a graph that describes the proportion of studies within each risk level per risk area.

Assessment of bias in conducting the systematic review

We will conduct the systematic review following this pre-specified protocol and report any differences between the methods of the complete review and this protocol in the review.

Data synthesis

Criteria for quantitative data synthesis

We plan to carry out a formal meta-analysis only where more than a single study per outcome is identified and the study designs, protocols and measures of treatment effect are considered similar enough to produce a meaningful pooled effect.

Measures of treatment effect

For dichotomous data, the treatment effect will be estimated and presented as a risk ratio with 95% confidence intervals. For time-to-event data, we will present the results as a log hazard ratio with its standard error. For studies reporting on serological response using a cohort design without a comparison group, we will present the effect of vaccination as the post-vaccination antibody level and the pre- to post-vaccination fold change in geometric mean antibody concentration.

Unit of analysis issues

The outcomes will be analysed at the level of study participants from each individual study.

For peer review only - http://bmjopen.bmj.com/site/about/guidelines.xhtml

Dealing with missing data

We will contact investigators to obtain numerical outcome data that have not been fully reported (for instance where when a study is identified as an abstract only or outcomes are reported in figures only). Where possible, we will calculate missing standard deviations from other reported statistics such as confidence intervals or standard errors. The impact of including studies with high levels of missing outcome data on the treatment effect will be explored in the sensitivity analysis.

Assessment of heterogeneity

To assess heterogeneity between studies, we plan to present a forest plot for each of the review outcomes. We will then calculate the formal heterogeneity variance statistics τ^2 (using the Restricted Maximum Likelihood Estimation [REML] method), I^2 and the Q-statistic. We will regard heterogeneity as substantial if τ^2 is greater than 0, I^2 is more than 30% and the p-value for Q-statistic is less than 0.10. We plan to further explore the potential causes of substantial heterogeneity in the subgroup analyses or meta-regression (specified below).

Quantitative data synthesis

Statistical analyses will be performed using Stata or R Studio. To account for the presence of heterogeneity, we will use random-effects meta-analysis to summarise the average effects of vaccination on the defined outcomes across studies. The results will be presented in forest plots with the average treatment effect (RR) with 95% confidence intervals, and the estimates of τ^2 and I^2. We will use the pooled average treatment effect to calculate the effectiveness of the vaccines (100*[1-RR]) in preventing the primary outcomes. The immunogenetic effect of the vaccines will be summarised as the mean post-vaccination antibody level with 95% confidence intervals and the mean pre- to post-vaccination fold change in geometric mean antibody concentration with 95% confidence

For peer review only - http://bmjopen.bmj.com/site/about/guidelines.xhtml

intervals. Observational studies and randomised controlled trial studies will be considered in separate analyses.

Subgroup analysis and investigation of heterogeneity

In case we identify an adequate number of studies (studies per explanatory variable ≥10) we plan to investigate the potential causes of heterogeneity between studies through random effects meta-regression analyses. We will consider the following categories of explanatory variables: severity of liver disease, aetiology of liver disease and the reason for hospital admission/ mortality (primary outcomes). Inclusion/exclusion of the explanatory variables in the heterogeneity investigations will depend on the characteristics and design of the identified studies. If we do not identify enough studies to perform meta-regressions but there are a minimum 5 studies per analysis we plan to carry out subgroup analyses to investigate whether these explanatory variables can explain heterogeneity between the studies.

Sensitivity analysis

In case the identified studies differ in terms of risk of bias, we plan to investigate the impact of excluding studies with high/unclear risk of bias on effect estimates in sensitivity analyses.

Qualitative data synthesis

We will provide a narrative summary of the study results for all outcomes, categorised by study design and vaccine type (influenza vaccine and pneumococcal vaccine). For the primary outcomes, we will report the cause of hospitalisation and death studied. Characteristics (participants, interventions, comparators, outcomes, study design and methods and notes on funding and conflicts of interests) of all studies included in the review will also be presented in separate tables. The results for outcomes where meta-analysis will not be

For peer review only

carried out due to insufficient homogeneity between studies will be presented in forest plots without the pooled effect estimate.

Meta-bias(es)

Assessment of reporting biases across studies

We plan to investigate reporting bias using funnel plots. If there are enough studies in the analysis (minimum 10), we will also carry out the Egger's test to assess whether there is a linear association between the study's result and its standard error.

We plan to assess selective outcome reporting bias by comparing what the study set to measure and analyse in the methods section of the study report (for studies published after 2006, we will also investigate the details trial protocol if it can be identified through the WHO International Clinical Trials Registry Platform,[21] launched in 2007) with the results that were reported. Using the Outcome Reporting Bias in Trials (ORBIT) classification system,[22] we will evaluate whether the risk of selective outcome reporting bias is present and whether the risk is low or high.

Confidence in cumulative evidence

We will use the Grading of Recommendations Assessment, Development and Evaluation (GRADE) Working Group system,[23] to assess and report the overall quality of the body of evidence for each outcome studied. The within-study risk of bias (methodological quality), directness of evidence, heterogeneity, the precision of effect estimates and risk of publication bias will be independently assessed by two review authors (SH&CP). The quality of evidence will be judged and reported as "high", "moderate", "low", or "very low" following the Cochrane Handbook for Systematic Reviews of Interventions guidelines,[15].

For peer review only

COMPETING INTERESTS

The authors declare no competing interests.

FUNDING

This work was supported by the UK Biotechnology and Biological Sciences Research Council grant number BBSRC BB/M009513/1.

AUTHOR CONTRIBUTIONS

The study was conceived by SH, LS, AO and AH. SH developed the eligibility criteria, search strategy, risk of bias assessment strategy and data extraction plan with guidance from LS, AO and AH. SH wrote the manuscript, to which all authors SH, CP, LS, AO and AH contributed.

ETHICS AND DISSEMINATION

Formal ethical approval is not required for this study, as no primary data will be collected. We will publish results of this study in relevant peer-reviewed medical journal or journals. Where possible, the study results will also be presented as posters or talks at relevant medical conferences and meetings.

PROTOCOL REGISTRATION

This systematic review protocol was registered in the International Prospective Register of Systematic Reviews (PROSPERO) on 13th June 2017 (registration number CRD42017067277).

For peer review only

REFERENCES

1 Gil-Prieto R, Pascual-Garcia R, Walter S, *et al.* Risk of hospitalization due to pneumococcal disease in adults in Spain. The CORIENNE study. *Hum Vaccin Immunother* 2016;**12**:1900–5. doi:10.1080/21645515.2016.1143577

2 Jan A, Hoek V, Andrews N, *et al.* The effect of underlying clinical conditions on the risk of developing invasive pneumococcal disease in England. *J Infect* 2012;**65**:17–24. doi:10.1016/j.jinf.2012.02.017

3 Puig-Barberà J, Natividad-Sancho A, Trushakova S, *et al.* Epidemiology of Hospital Admissions with Influenza during the 2013 / 2014 Northern Hemisphere Influenza Season: Results from the Global Influenza Hospital Surveillance Network. *PLoS One* 2016;**11**:e0154970. doi:10.1371/journal.pone.0154970

4 Kerkhove MD Van, Vandemaele KAH, Shinde V, *et al.* Risk Factors for Severe Outcomes following 2009 Influenza A (H1N1) Infection: A Global Pooled Analysis. *PLoS Med* 2011;**8**:e1001053. doi:10.1371/journal.pmed.1001053

5 Polakos NK, Cornejo JC, Murray DA, *et al.* Kupffer Cell-Dependent Hepatitis Occurs during Influenza Infection. *Am J Pathol* 2006;**168**:1169–78. doi:10.2353/ajpath.2006.050875

6 Duchini A, Viernes ME, Nyberg LM, *et al.* Hepatic Decompensation in Patients With Cirrhosis During Infection With Influenza A. *Arch Intern Med* 2000;**160**:113–5. doi:10.1001/archinte.160.1.113

7 World Health Organization. Weekly epidemiological record: Vaccines against influenza. WHO position paper. 2012;:461–76.http://www.who.int/wer/2012/wer8747/en/ (accessed 13 Jun 2017).

8 Kanitz EE, Wu LA, Giambi C, *et al.* Variation in adult vaccination policies across Europe: An overview from VENICE network on vaccine recommendations, funding and coverage. *Vaccine* 2012;**30**:5222–8. doi:10.1016/j.vaccine.2012.06.012

9 European Centre for Disease Prevention and Control. Seasonal influenza vaccination and antiviral use in Europe – Overview of vaccination

For peer review only - http://bmjopen.bmj.com/site/about/guidelines.xhtml

recommendations and coverage rates in the EU Member States for the 2013–14 and 2014–15 influenza seasons. 2016.http://ecdc.europa.eu/en/publications/_layouts/forms/Publication_DispForm.aspx?List=4f55ad51-4aed-4d32-b960-af70113dbb90&ID=1528 (accessed 13 Jun 2017).

10 Pebody RG, Leino T, Nohynek H, *et al.* Pneumococcal vaccination policy in Europe. Eurosurveillance. 2005;**10**.http://www.eurosurveillance.org/ViewArticle.aspx?ArticleId=564 (accessed 13 Jun 2017).

11 Public Health England. Influenza immunisation programme for England: GP patient groups - Data collection survey Season 2015 to 2016. 2016.https://www.gov.uk/government/statistics/seasonal-flu-vaccine-uptake-in-gp-patients-in-england-winter-season-2015-to-2016 (accessed 13 Jun 2017).

12 Blachier M, Leleu H, Peck-Radosavljevic M, *et al.* The burden of liver disease in Europe: a review of available epidemiological data. *J Hepatol* 2013;**58**:593–608. doi:10.1016/j.jhep.2012.12.005

13 Petticrew M, Shekelle P, Stewart LA, *et al.* Preferred reporting items for systematic review and meta-analysis protocols (PRISMA-P) 2015: elaboration and explanation. *BMJ* 2015;**349**:g7647. doi:10.1136/bmj.g7647

14 Kastner M, Wilczynski NL, Walker-Dilks C, *et al.* Age-Specific Search Strategies for Medline. *J Med Internet Res* 2006;**8**:e25. doi:10.2196/jmir.8.4.e25

15 The Cochrane Collaboration. Cochrane Handbook for Systematic Reviews of Interventions. 2011.www.handbook.cochrane.org (accessed 13 Jun 2017).

16 Chalmers JD, Campling J, Dicker A, *et al.* A systematic review of the burden of vaccine preventable pneumococcal disease in UK adults. *BMC Pulm Med* 2016;:1–11. doi:10.1186/s12890-016-0242-0

17 University of Texas School of Public Health. Search filters for case-control studies, cohort studies, cross-sectional studies, clinical trials. 2012.http://libguides.sph.uth.tmc.edu/search_filters/ovid_psycinfo_filters

For peer review only

(accessed 13 Jun 2017).

18 Scottish Intercollegiate Guidelines Network. SIGN search filters for observational studies.

19 BMJ Evidence Centre. Medline cohort and case-control strategy. 2012.http://clinicalevidence.bmj.com/x/set/static/ebm/learn/665076.html (accessed 13 Jun 2017).

20 Moher D, Liberati A, Tetzlaff J, *et al.* Academia and Clinic Annals of Internal Medicine Preferred Reporting Items for Systematic Reviews and Meta-Analyses: The PRISMA Statement. *Annu Intern Med* 2009;**151**:264–9. doi:10.1371/journal.pmed1000097

21 World Health Organization. International Clinical Trials Registry Platform Search Portal. http://apps.who.int/trialsearch/ (accessed 13 Jun 2017).

22 Kirkham JJ, Dwan KM, Altman DG, *et al.* The impact of outcome reporting bias in randomised controlled trials on a cohort of systematic reviews. *BMJ* 2010;**340**:c365. doi:10.1136/bmj.c365

23 Guyatt GH, Oxman AD, Vist GE, *et al.* GRADE: an emerging consensus on rating quality of evidence and strength of recommendations. *BMJ* 2008;**336**:924–6. doi:10.1136/bmj.39489.470347.AD

For peer review only

PRISMA-P (Preferred Reporting Items for Systematic review and Meta-Analysis Protocols) 2015 checklist: recommended items to address in a systematic review protocol*

Section and topic	Item No	Checklist item	Status	Page
**ADMINISTRATIVE INFORMATION**				
Title:				
Identification	1a	Identify the report as a protocol of a systematic review	Completed	1
Update	1b	If the protocol is for an update of a previous systematic review, identify as such	n/a	n/a
Registration	2	If registered, provide the name of the registry (such as PROSPERO) and registration number	Completed	2, 21
Authors:				
Contact	3a	Provide name, institutional affiliation, e-mail address of all protocol authors; provide physical mailing address of corresponding author	Completed	1
Contributions	3b	Describe contributions of protocol authors and identify the guarantor of the review	Completed	21
Amendments	4	If the protocol represents an amendment of a previously completed or published protocol, identify as such and list changes; otherwise, state plan for documenting important protocol amendments	Completed	17
Support:				
Sources	5a	Indicate sources of financial or other support for the review	Completed	21
Sponsor	5b	Provide name for the review funder and/or sponsor	Completed	21
Role of sponsor or funder	5c	Describe roles of funder(s), sponsor(s), and/or institution(s), if any, in developing the protocol	Completed	21
**INTRODUCTION**				
Rationale	6	Describe the rationale for the review in the	Completed	4-6

For peer review only

		context of what is already known		
Objectives	7	Provide an explicit statement of the question(s) the review will address with reference to participants, interventions, comparators, and outcomes (PICO)	Completed	7
**METHODS**				
Eligibility criteria	8	Specify the study characteristics (such as PICO, study design, setting, time frame) and report characteristics (such as years considered, language, publication status) to be used as criteria for eligibility for the review	Completed	7-9
Information sources	9	Describe all intended information sources (such as electronic databases, contact with study authors, trial registers or other grey literature sources) with planned dates of coverage	Completed	9
Search strategy	10	Present draft of search strategy to be used for at least one electronic database, including planned limits, such that it could be repeated	Completed	9-12
Study records:				
Data management	11a	Describe the mechanism(s) that will be used to manage records and data throughout the review	Completed	13
Selection process	11b	State the process that will be used for selecting studies (such as two independent reviewers) through each phase of the review (that is, screening, eligibility and inclusion in meta-analysis)	Completed	13
Data collection process	11c	Describe planned method of extracting data from reports (such as piloting forms, done independently, in duplicate), any processes for obtaining and confirming data from investigators	Completed	13-14
Data items	12	List and define all variables for which data will be sought (such as PICO items, funding sources), any pre-planned data assumptions and simplifications	Completed	14-15
Outcomes and	13	List and define all outcomes for which data will	Completed	15-16

For peer review only

prioritization		be sought, including prioritization of main and additional outcomes, with rationale		
Risk of bias in individual studies	14	Describe anticipated methods for assessing risk of bias of individual studies, including whether this will be done at the outcome or study level, or both; state how this information will be used in data synthesis	Completed	16-17
Data synthesis	15a	Describe criteria under which study data will be quantitatively synthesised	Completed	17
	15b	If data are appropriate for quantitative synthesis, describe planned summary measures, methods of handling data and methods of combining data from studies, including any planned exploration of consistency (such as I^2, Kendall's τ)	Completed	17-19
	15c	Describe any proposed additional analyses (such as sensitivity or subgroup analyses, meta-regression)	Completed	19
	15d	If quantitative synthesis is not appropriate, describe the type of summary planned	Completed	19-20
Meta-bias(es)	16	Specify any planned assessment of meta-bias(es) (such as publication bias across studies, selective reporting within studies)	Completed	20
Confidence in cumulative evidence	17	Describe how the strength of the body of evidence will be assessed (such as GRADE)	Completed	20

*** It is strongly recommended that this checklist be read in conjunction with the PRISMA-P Explanation and Elaboration (cite when available) for important clarification on the items. Amendments to a review protocol should be tracked and dated. The copyright for PRISMA-P (including checklist) is held by the PRISMA-P Group and is distributed under a Creative Commons Attribution Licence 4.0.**

From: Shamseer L, Moher D, Clarke M, Ghersi D, Liberati A, Petticrew M, Shekelle P, Stewart L, PRISMA-P Group. Preferred reporting items for systematic review and meta-analysis protocols (PRISMA-P) 2015: elaboration and explanation. BMJ. 2015 Jan 2;349(jan02 1):g7647.

For peer review only

BMJ Open

EFFECTIVENESS OF PNEUMOCOCCAL AND INFLUENZA VACCINES TO PREVENT SERIOUS HEALTH COMPLICATIONS IN ADULTS WITH CHRONIC LIVER DISEASE: A PROTOCOL FOR A SYSTEMATIC REVIEW

Journal:	*BMJ Open*
Manuscript ID	bmjopen-2017-018223.R1
Article Type:	Protocol
Date Submitted by the Author:	21-Dec-2017

Complete List of Authors:	Härmälä, Suvi; University College London, Institute of Health Informatics<br>Parisinos, Constantinos; University College London, Institute of Health Informatics<br>Shallcross, Laura; University College London, Institute of Health Informatics<br>O'Brien, Alastair; University College London, Division of Medicine<br>Hayward, Andrew; University College London, Institute of Epidemiology and Health Care

**Primary Subject Heading**:	Infectious diseases
Secondary Subject Heading:	Gastroenterology and hepatology, Public health
Keywords:	Pneumococcal vaccine, Influenza vaccine, Chronic liver disease

SCHOLARONE™ Manuscripts

For peer review only

EFFECTIVENESS OF PNEUMOCOCCAL AND INFLUENZA VACCINES TO PREVENT SERIOUS HEALTH COMPLICATIONS IN ADULTS WITH CHRONIC LIVER DISEASE: A PROTOCOL FOR A SYSTEMATIC REVIEW

Suvi Härmälä[1], Constantinos Parisinos[1], Laura Shallcross[1], Alastair O'Brien[2] & Andrew Hayward[1,3]

[1] Institute of Health Informatics, University College London, London, United Kingdom.

[2] Division of Medicine, University College London, London, United Kingdom.

[3] Institute of Epidemiology and Health Care, University College London, London, United Kingdom.

Corresponding author: Suvi K. Härmälä, Institute of Health Informatics, University College London, 222 Euston Road, London NW1 2DA, United Kingdom, suvi.harmala.14@ucl.ac.uk, +44 (0)20 3549 5338

Keywords: Pneumococcal vaccine, influenza vaccine, chronic liver disease

Word count (excluding title page, abstract, strengths & limitations, references and table): 3909

For peer review only

ABSTRACT

Introduction: In advanced chronic liver disease, diseases caused by common bacteria *Streptococcus pneumoniae* or influenza virus put people at an increased risk of serious health complications and death. The effectiveness of the available vaccines in reducing the risk of poor health outcomes, however, is less clear.

Methods and analysis: We will search MEDLINE (Ovid), EMBASE (Ovid), Pubmed and Cochrane Central Register of Controlled Trials (CENTRAL) for published reports on randomised controlled trials and observational studies on the effectiveness of pneumococcal and influenza vaccines in people with chronic liver disease. Two independent reviewers will screen the studies for eligibility, extract data and assess study quality and risk of bias. Random effects meta-analyses will be performed as appropriate.

Ethics and dissemination: Formal ethical approval is not required, as no primary data will be collected for this study. We will publish results of this study in relevant peer-reviewed medical journal or journals. Where possible, the study results will also be presented as posters or talks at relevant medical conferences and meetings.

Prospero registration number: CRD42017067277

For peer review only

STRENGTHS AND LIMITATIONS OF THIS STUDY:

- This study protocol follows the recommendations by the Preferred Reporting Items for Systematic Review and Meta-Analysis Protocols (PRISMA-P).
- This study protocol has been prospectively registered in the International Prospective Register of Systematic Reviews (PROSPERO).
- Our comprehensive search strategy will minimise the risk of missing relevant studies, in particular, those with a randomised design.
- The selection of studies, data extraction, the risk of bias and quality of evidence assessments will be conducted by two independent authors.
- Inclusion of studies with a non-randomised design may decrease the overall quality of the body of evidence for the study outcomes.

For peer review only

BACKGROUND

Burden of pneumococcal and influenza infections in chronic liver disease

In advanced chronic liver disease, as the immune function progressively deteriorates, diseases caused by common bacteria *Streptococcus pneumoniae* or influenza virus can lead to serious health complications and death.

In Spain in 2011, the population-level annual incidence rate for pneumococcal pneumonia-related hospitalisation in adults with liver disease was estimated at approximately 540 per 100 000 compared to approximately 6 per 100 000 without at-risk conditions,[1]. Adults with liver disease were over 50 times more likely to be hospitalised for pneumococcal pneumonia than adults without at-risk conditions,[1]. Similarly, in England in 2008/2009, for invasive pneumococcal disease (IPD), such as meningitis, bacteraemia and sepsis, the annual incidence of hospitalisation in adults with liver disease was estimated at about 100 per 100 000 compared to about 8 per 100 000 in the healthy population,[2]. Approximately 37% of liver disease patients hospitalised for IPD died, compared to 5% of patients without underlying risk conditions,[2]. Chronic liver disease patients were over 30 times more likely to be admitted to hospital and 10 times more likely to die during the IPD-related hospitalisation than adults without at-risk conditions,[2].

Although there are no population-level estimates of severe influenza incidence in people with chronic liver disease, evidence suggests that liver disease patients are at an increased risk of health complications from influenza. A 2-fold increased risk of influenza admission was observed in liver disease patients at 19 hospitals in Russia, Turkey, China, and Spain during the 2013/2014 season,[3]. Similarly, an analysis of data on laboratory-confirmed influenza cases collected from several World Health Organisation (WHO) member states during the 2009 influenza A (H1N1) pandemic found liver disease patients to have a greater than 5-fold increased risk of influenza-related hospitalisation and over 17-fold increased risk of death compared to that of healthy individuals,[4].

For peer review only - http://bmjopen.bmj.com/site/about/guidelines.xhtml

Furthermore, influenza infection, while not directly targeting the liver, may cause collateral transient liver damage,[5] and trigger hepatic decompensation (such as ascites and hepatic encephalopathy) in liver disease patients,[6].

Types of pneumococcal and influenza vaccines, vaccination policy and vaccine uptake

Two types of vaccines, polysaccharide pneumococcal vaccines including serotypes of *S. pneumoniae* (PPV23) and conjugate pneumococcal vaccines including 7 (PCV7), 10 (PCV10) or 13 (PCV13) *S. pneumoniae* serotypes, are available to protect against pneumococcal infection. None of these contains live bacteria.The most commonly used influenza vaccines are injectable, inactivated vaccines that contain either inactivated whole influenza virus or split or subunit virus products. These vaccines protect either against two influenza A (H1N1 and H3N2) strains and one influenza B strain (trivalent vaccines) or two influenza A and two influenza B strains (quadrivalent vaccines). New vaccines are developed every year to protect against the prevailing strains of the upcoming influenza season and a yearly vaccination is recommended to ensure continued protection. Live attenuated influenza vaccines exist, however, these may not be suitable for people with chronic co-morbidities,[7]. Immune response defects associated with advanced liver disease, [8–11] may also dampen the response to vaccines.

The majority of European countries recommend both adult influenza (29/29 countries) and pneumococcal vaccination (22/29 countries) for specific high-risk groups,[12]. Chronic liver disease patients, however, may not be included in these high-risk target groups in all countries. Whilst 90% (27/ 30) of the countries recommended influenza immunisation for people with liver disease during the 2014-2015 influenza season,[13], only 43% (6/14) of countries surveyed in 2005 recommended pneumococcal vaccination for chronic liver disease patients,[14]. Moreover, whilst the adult vaccination recommendations for influenza are supported by official funding mechanisms in most countries (21/29), the cost of the pneumococcal vaccination is covered by only half (11/22),[12].

For peer review only

Uptake of influenza vaccine in people with chronic diseases in general is poor. The median coverage rate across Europe in the 2014/2015 season was less than 50% (only 7/30 countries were able to provide separate coverage data for individuals with chronic medical conditions),[13]. The situation in liver disease patients as a separate group seems no different with less than 50% of working-age liver disease patients in the UK covered by the influenza vaccine in 2015/2016 season,[15]. Although fewer data exist concerning the uptake of pneumococcal adult vaccination,[12], its uptake in liver disease patients is not likely to be any higher than that of influenza vaccine.

Rationale for the review

Approximately 29 million people in Europe alone are affected by some form of liver disease,[16]. Worryingly, due to the increasing prevalence of obesity and persistence of other liver damage risk factors such as alcohol abuse and hepatitis infections, this number is expected to grow,[16]. Whilst evidence suggests that following infection with influenza or *S. pneumoniae*, people with liver disease have a higher than average risk of poor health outcomes, the effectiveness of the vaccines in reducing this risk is less clear and warrants further investigation.

To our knowledge, no systematic review to date has investigated the effects of pneumococcal and influenza vaccines in preventing poor health outcomes in chronic liver disease. The present review intends to fill this gap by providing a systematic synthesis of the available evidence. The results of this review may inform future vaccination strategies and help improve vaccination coverage. This, in turn, may have a positive impact on both the number of influenza and pneumococcal disease-related hospital admissions and patient outcomes in liver disease.

OBJECTIVES

For peer review only - http://bmjopen.bmj.com/site/about/guidelines.xhtml

The aim of this review is to assess the effectiveness of pneumococcal and influenza vaccines to prevent serious health complications in adults with chronic liver disease.

The objectives are:

- To assess the effectiveness of pneumococcal and influenza vaccines to prevent hospitalisation in adults with chronic liver disease.
- To assess the effectiveness of pneumococcal and influenza vaccines to prevent death in adults with chronic liver disease.
- To assess the effects of pneumococcal and influenza vaccines for eliciting a serological response in adults with chronic liver disease.

METHODS

This study protocol follows the recommendations by the Preferred Reporting Items for Systematic Review and Meta-Analysis Protocols (PRISMA-P) 2015,[17].

Eligibility criteria

Types of studies

We will include all randomized clinical trials, cohort (with comparison group/s) and case-control studies that investigate the effectiveness of pneumococcal or influenza vaccines for preventing hospitalisation or death in adults with chronic liver disease. We will also include all randomized clinical trials, cohort (with or without comparison group/s) and case-control studies that report the serological response to one or both of these vaccines in adults with chronic liver disease. We will only include published studies in English language and studies that have been published or accepted for publication. We will exclude review articles, case reports, cross-sectional studies, animal studies, editorials, clinical guidelines and any studies that have been fully or partially retracted from publication. Patients included in multiple studies will be reported only once.

For peer review only

Types of participants

We will include studies that enrol 18+ year-old adult patients with chronic liver disease of any severity (non-cirrhotic, cirrhotic) or aetiology (viral, alcoholic, non-alcoholic fatty liver, autoimmune).

Types of interventions

We will include studies that investigate the effects of a conjugate or polysaccharide pneumococcal vaccine (against *S. pneumoniae*) and/or an inactivated (whole virus, split virus or subunit), injectable influenza vaccine. Vaccines can be adjuvanted, intradermal and of any dose. We will exclude live, recombinant, virosomal and experimental vaccines.

Types of comparators

We will include studies comparing one or both of the vaccines of interest to a placebo, an alternative intervention or no intervention. We will also include studies without a comparison group when the outcome studied is the serological response to the vaccine.

Types of outcome measures

We will include studies that report on one or more of our primary outcomes and/or our secondary outcome of interest.

Primary outcomes

- All-cause hospitalisation
- All-cause mortality

Secondary outcomes

- Serological response to vaccine

For peer review only

- Acute respiratory illness-related hospitalisation
- Influenza illness or influenza-like-illness (ILI)-related hospitalisation
- Pneumococcal disease-related hospitalisation
- Hospitalisation for liver disease complications (variceal bleeding, hepatic encephalopathy, ascites, spontaneous bacterial peritonitis, jaundice and bacteraemia or sepsis)
- Acute respiratory illness-related mortality
- Influenza illness or ILI-related mortality
- Pneumococcal disease-related mortality
- Liver disease-related mortality

Information sources

Electronic searches

To capture all relevant studies, we plan to search the following databases:

- Cochrane Central Register of Controlled Trials (CENTRAL)
- MEDLINE (Ovid)
- EMBASE (Ovid)
- PubMed
-

Each database will be searched separately and the search strategy first developed in MEDLINE will be adapted to each database interface as appropriate. We plan to also search relevant studies from the reference lists of the eligible studies identified through the electronic searches.

Search strategy

We will use two complementary strategies to identify relevant articles. First, we will identify articles reporting outcomes of vaccination in patients with liver disease by combining search terms for influenza and pneumococcal vaccination with search terms for chronic liver disease (Search 1). This search will be

For peer review only

filtered by study design. The provisional search terms for liver disease and pneumococcal and influenza vaccines are listed in Table 1.

Recognising that liver disease patients may be included as subgroups in clinical trials of vaccination, we will also search for randomised controlled trials of influenza and pneumococcal vaccine that have recruited individuals from the general population (Search 2).

To search for studies with adult participants, we will combine the geriatric and adult medicine-specific search strategies by Kastner et al.,[18]. In order to maximise the sensitivity of the searches for randomised clinical trials, we will use a filter that combines terms from the Cochrane Highly Sensitive Search Strategy,[19] and less specific version of the same filter by Chalmers et al.,[20] to identify randomised trials. Similarly, to maximise the sensitivity of searches for case-control and cohort studies, we will use a filter that combines terms from the University of Texas School of Public Health filter for observational studies,[21], SIGN observational study filter,[22] and the BMJ Evidence Centre case-control and cohort strategy,[23]. We will limit our search to studies including adult human study subjects but not based on the setting/country or publication year of the articles. The search terms for adult and study design filters are listed in Table 1.

Table 1. MEDLINE (Ovid) provisional search terms

Search concept	Search terms
Pneumococcal vaccine	1. exp Pneumococcal Vaccines/
	2. exp Pneumococcal Infections/pc [Prevention & Control]
	3. ((anti?pneum* or pneum*) adj5 (vaccin* or immuni*)).mp.
	4. (PPV?23* or PPSV or PPSV?23* or PCV?7* or PCV?10* or PCV?13*).mp.
	5. ((PPV or PCV) adj5 (pneum* or vaccin* or immuni*)).mp.
	6. ((7?valent or hepta?valent or 10?valent or 13?valent or 23?valent) adj5 (vaccin* or immuni*)).mp.

	7. 1 or 2 or 3 or 4 or 5 or 6 or 7
Influenza vaccine	1. Influenza Vaccines/
	2. Influenza, Human/pc [Prevention & Control]
	3. ((anti?influenza or influenza or seasonal or anti?flu or flu) adj5 (vaccin* or immuni*)).mp.
	4. ((TIV or QIV or trivalent or quadrivalent or 3?valent or 4?valent) adj5 (vaccin* or immuni*)).mp.
	5. 1 or 2 or 3 or 4 or 5
Liver disease	1. exp Liver Diseases/
	2. ((liver or hepat*) adj3 disease*).mp.
	3. ("chronic liver" or "chronic hepat*").mp.
	4. cirrho*.mp.
	5. 1 or 2 or 3 or 4
Adult participants	1. exp Adult/
	2. adult.mp.
	3. (middle?aged or aged).sh.
	4. age*.tw.
	5. 1 or 2 or 3 or 4
Randomised controlled trials	1. randomized controlled trial.pt.
	2. randomi*.ab,ti.
	3. randomly.ab,ti.
	4. controlled clinical trial.pt.
	5. trial.ab,ti.
	6. groups.ab,ti.
	7. drug therapy.fs.
	8. placebo.ab,ti.
	9. 1 or 2 or3 or 4 or 5 or 6 or 7 or 8
	10. Animals/
	11. Humans/

For peer review only

	12. 10 not (10 and 11)
	13. 9 not 12
Case-control and cohort studies	1. Epidemiologic Studies/
	2. exp Case control studies/
	3. exp Cohort studies/
	4. Longitudinal studies/
	5. Follow up studies/
	6. Prospective studies/
	7. Retrospective studies/
	8. Control groups/
	9. Matched-Pair Analysis/
	10. (Case* adj5 control*).ti,ab,kw.
	11. (Case* adj5 comparison*).ti,ab,kw.
	12. Control group*.ti,ab,kw.
	13. (Cohort adj (study or studies)).ti,ab.
	14. Cohort anal*.ti,ab.
	15. (Follow up adj (study or studies)).ti,ab.
	16. (Observational adj (study or studies)).ti,ab.
	17. Longitudinal.ti,ab.
	18. Retrospective.ti,ab.
	19. Prospective.ti,ab.
	20. 1 or 2 or 3 or 4 or 5 or 6 or 7 or 8 or 9 or 10 or 11 or 12 or 13 or 14 or 15 or 16 or 17 or 18 or 19
	21. Animals/
	22. Humans/
	23. 21 not (21 and 22)
	24. 20 not 23

Study records

For peer review only

Data management

The search results will be uploaded into reference management software (Mendeley) to remove duplicate records of the same report. The unique records will then be uploaded into web-based, systematic review management software (DistillerSR). Both the initial abstract and title screening and the full-text review and extraction of data from the eligible studies will be performed using standardised, pre-created online forms. All forms will be piloted and revised as needed by the reviewers before starting the review.

Selection process

Articles that have been identified through the broad search of RCTs of pneumococcal or flu vaccination in the general population (Search 2) will first be pre-screened by title by one reviewer (SH). Articles meeting pre-screening criteria will be combined with the articles identified through Search 1. These articles will then all be screened by two independent reviewers (SH&CP) by abstract and title. Where the study eligibility cannot be established based on the title and abstract, the report will be passed on to the full-text review. Similarly, records subject to disagreement over eligibility will be included in the full-text review.

The full-text review will be independently completed for all eligible articles by two independent reviewers authors (SH&CP). Reasons for exclusion of ineligible studies will be recorded. Disagreements will be resolved by consulting a third review author (AO) and any uncertainties by correspondence with study investigators. Multiple reports of the same study will be collated into one and, where not possible, only the most relevant report based on our eligibility criteria will be included. The study selection process will be recorded and presented in flow diagram format according to the recommendations of Preferred Reporting Items for Systematic Reviews and Meta-Analyses (PRISMA),[24].

Data collection process

For peer review only

The data will be extracted and entered into standardised, pre-created online data extraction forms independently and in duplicate by two review authors (SH&CP). Disagreement will be resolved by consulting a third review author (LS) and uncertainties by correspondence with study investigators.

Data items

We will extract data on:

- Study participants: inclusion and exclusion criteria, method of recruitment/selection, study population characteristics and any imbalances at baseline (sex, age, aetiology and severity of liver disease, co-morbidities, alcohol use, smoking status, pre-vaccination infection status, medication/treatment other than intervention).
- Interventions and comparators (vaccine type, comparison treatment, dose, route of delivery, number and timing of vaccinations/comparator treatments, number of individuals in intervention and comparison group, follow-up time in intervention and comparison groups)
- Outcomes (definition, time points measured and reported, unit of measurement, number of outcomes in the intervention and control group, unadjusted and adjusted effect measures, covariates that the effect measures were adjusted for, comparisons, missing data and reasons for missingness, statistical methods used, processes for randomisation e.g. allocation concealment)
- Study designs and methods (study type, country and setting, date of study, study duration, aim of study, withdrawals).
- Study quality and study bias (according to the needs of the assessments specified below).
- Study funding and conflicts of interest

Effect measures will be collected in the format in which they are reported and transformed for presentation and analysis if appropriate.

For peer review only

Outcomes and prioritisation

Our main outcomes of interest are hospitalisation and death. These are potential severe outcomes of influenza illness and pneumococcal disease. It may be challenging to identify and establish, especially if hospital discharge records are reviewed retrospectively, the exact cause of the hospitalisation or death of a patient with an underlying chronic condition. For this reason, our primary outcomes we will include all causes. Additionally, studies may not always specify whether a patient with a diagnosis of an infectious disease or liver disease complication was actually hospitalised. We will assume that when a patient during the follow-up after the vaccination developed any liver disease complication (variceal bleeding, hepatic encephalopathy, ascites, jaundice, spontaneous bacterial peritonitis or bacteraemia or sepsis) or was diagnosed with IPD, they also required hospitalisation. Acute respiratory illness, non-invasive pneumococcal disease and influenza illness or ILI may not require hospitalisation so unless it is specified that the patient was hospitalised or the illness was recorded in the hospital records, we will assume the patient was not hospitalised.

Our secondary outcomes of interest are the serological response to pneumococcal and influenza vaccines and a range of cause-specific hospitalisation and mortality. Serological response to a vaccine is an indicator of the vaccine's effect on building protective immunity against the disease the vaccination targets. Since, however, it is difficult to know what level of antibody or increase in antibody concentration may provide protection in people with chronic liver disease, we will evaluate both the post-vaccination antibody level and the pre- to post-vaccination fold change in geometric mean antibody concentrations. This effect will be evaluated both for short-term (<6 months) and long-term (≥6 months) after vaccination. In case antibody responses are reported at multiple short-term or long-term time points, we will consider the time point closest to the timing of vaccination as the as the short-term response and the time point closest to 6 months as the long-term response. The more

For peer review only - http://bmjopen.bmj.com/site/about/guidelines.xhtml

detailed causes of hospitalisation and death, allow us to understand about the more specific effects of the vaccines.

Assessment of risk of bias in individual studies

We will use the Cochrane Collaborations tool,[19] for assessing the risk of bias in all studies included in the review after the full-text review. The level of risk of bias in random sequence generation, allocation concealment, blinding of participants and personnel, blinding of outcome assessment, incomplete outcome data, selective reporting and other sources will be judged as “low”, “high” or “unclear” according to the criteria specified in the Cochrane Handbook,[19].

In the non-randomised studies, we will additionally assess the level of risk of confounding bias due to inadequately addressed differences between groups (i.e. is the effect estimate likely to be biased due to unaccounted confounding). We will consider age, sex, severity and aetiology of liver disease to be the most important potential confounders. We will judge the risk of confounding bias to be low if the study addressed the presence of these confounders by restricting participant selection by confounders, demonstrating balance between groups, matching on the confounders or adjusting for the confounders in statistical analyses of the effect size. The risk of confounding bias will be judged low if the confounders were adjusted for, high if the presence of the confounders was not addressed and unclear if there was insufficient information to judge the level of risk.

Two review authors (SH&CP) will independently assess the studies for each of the risk areas by entering a quote from the study to describe the procedures, their judgement together with a justification of the judgement into pre-created online forms in DistillerSR. Disagreements will be resolved by consulting a third review author (AH). The risk of bias assessments will be presented in a figure that shows the level of risk in the different risk areas within each individual

For peer review only

study and in a graph that describes the proportion of studies within each risk level per risk area.

Assessment of bias in conducting the systematic review

We will conduct the systematic review following this pre-specified protocol and report any differences between the methods of the complete review and this protocol in the review.

Data synthesis

Criteria for quantitative data synthesis

We plan to carry out a formal meta-analysis only where more than a single study per outcome is identified and the study designs, protocols and measures of treatment effect are considered similar enough to produce a meaningful pooled effect.

Measures of treatment effect

For dichotomous data, the treatment effect will be estimated and presented as a risk ratio with 95% confidence intervals. For time-to-event data, we will present the results as a log hazard ratio with its standard error. For studies reporting on serological response using a cohort design without a comparison group, we will present the effect of vaccination as the post-vaccination antibody level and the pre- to post-vaccination fold change in geometric mean antibody concentration.

Unit of analysis issues

The outcomes will be analysed at the level of study participants from each individual study.

Dealing with missing data

For peer review only - http://bmjopen.bmj.com/site/about/guidelines.xhtml

We will contact investigators to obtain numerical outcome data that have not been fully reported (for instance where when a study is identified as an abstract only or outcomes are reported in figures only). Where possible, we will calculate missing standard deviations from other reported statistics such as confidence intervals or standard errors. The impact of including studies with high levels of missing outcome data on the treatment effect will be explored in the sensitivity analysis.

Assessment of heterogeneity

To assess heterogeneity between studies, we plan to present a forest plot for each of the review outcomes. We will then calculate the formal heterogeneity variance statistics τ^2, I^2 and the Q-statistic. We will regard heterogeneity as substantial if τ^2 is greater than 0, I^2 is more than 30% and the p-value for Q-statistic is less than 0.10. We plan to further explore the potential causes of substantial heterogeneity in the subgroup analyses or meta-regression (specified below).

Quantitative data synthesis

Statistical analyses will be performed using Stata or R Studio. To account for the presence of heterogeneity, we will use random-effects meta-analysis to summarise the average effects of vaccination on the defined outcomes across studies. The results will be presented in forest plots with the average treatment effect (RR) with 95% confidence intervals, and the estimates of τ^2 and I^2. We will use the pooled average treatment effect to calculate the effectiveness of the vaccines (100*[1-RR]) in preventing the primary outcomes. The immunogenetic effect of the vaccines will be summarised as the mean post-vaccination antibody level with 95% confidence intervals and the mean pre- to post-vaccination fold change in geometric mean antibody concentration with 95% confidence intervals. Observational studies and randomised controlled trial studies will be considered in separate analyses.

For peer review only - http://bmjopen.bmj.com/site/about/guidelines.xhtml

Subgroup analysis and investigation of heterogeneity

In case we identify an adequate number of studies (studies per explanatory variable ≥10) we plan to investigate the potential causes of heterogeneity between studies through random effects meta-regression analyses. We will consider the following categories of explanatory variables: severity of liver disease, aetiology of liver disease and the reason for hospital admission/ mortality (primary outcomes). Inclusion/exclusion of the explanatory variables in the heterogeneity investigations will depend on the characteristics and design of the identified studies. If we do not identify enough studies to perform meta-regressions but there are a minimum 5 studies per analysis we plan to carry out subgroup analyses to investigate whether these explanatory variables can explain heterogeneity between the studies.

Sensitivity analysis

In case the identified studies differ in terms of risk of bias, we plan to investigate the impact of excluding studies with high/unclear risk of bias on effect estimates in sensitivity analyses.

Qualitative data synthesis

We will provide a narrative summary of the study results for all outcomes, categorised by study design and vaccine type (influenza vaccine and pneumococcal vaccine). For the primary outcomes, we will report the cause of hospitalisation and death studied. Characteristics (participants, interventions, comparators, outcomes, study design and methods and notes on funding and conflicts of interests) of all studies included in the review will also be presented in separate tables. The results for outcomes where meta-analysis will not be carried out due to insufficient homogeneity between studies will be presented in forest plots without the pooled effect estimate.

For peer review only

Meta-bias(es)

Assessment of reporting biases across studies

We plan to investigate reporting bias using funnel plots. If there are enough studies in the analysis (minimum 10), we will also carry out the Egger's test to assess whether there is a linear association between the study's result and its standard error.

We plan to assess selective outcome reporting bias by comparing what the study set to measure and analyse in the methods section of the study report (for studies published after 2006, we will also investigate the details trial protocol if it can be identified through the WHO International Clinical Trials Registry Platform,[25] launched in 2007) with the results that were reported. Using the Outcome Reporting Bias in Trials (ORBIT) classification system,[26] we will evaluate whether the risk of selective outcome reporting bias is present and whether the risk is low or high.

Confidence in cumulative evidence

We will use the Grading of Recommendations Assessment, Development and Evaluation (GRADE) Working Group system,[27] to assess and report the overall quality of the body of evidence for each outcome studied. The within-study risk of bias (methodological quality), directness of evidence, heterogeneity, the precision of effect estimates and risk of publication bias will be independently assessed by two review authors (SH&CP). The quality of evidence will be judged and reported as "high", "moderate", "low", or "very low" following the Cochrane Handbook for Systematic Reviews of Interventions guidelines,[19].

COMPETING INTERESTS

The authors declare no competing interests.

For peer review only - http://bmjopen.bmj.com/site/about/guidelines.xhtml

FUNDING

This work was supported by the UK Biotechnology and Biological Sciences Research Council grant number BBSRC BB/M009513/1 to SH.

AUTHOR CONTRIBUTIONS

The study was conceived by SH, LS, AO and AH. SH developed the eligibility criteria, search strategy, risk of bias assessment strategy and data extraction plan with guidance from LS, AO and AH. SH wrote the manuscript, to which all authors SH, CP, LS, AO and AH contributed.

ETHICS AND DISSEMINATION

Formal ethical approval is not required for this study, as no primary data will be collected. We will publish results of this study in relevant peer-reviewed medical journal or journals. Where possible, the study results will also be presented as posters or talks at relevant medical conferences and meetings.

PROTOCOL REGISTRATION

This systematic review protocol was registered in the International Prospective Register of Systematic Reviews (PROSPERO) on 13th June 2017 (registration number CRD42017067277).

REFERENCES

1 Gil-Prieto R, Pascual-Garcia R, Walter S, *et al.* Risk of hospitalization due to

For peer review only

pneumococcal disease in adults in Spain. The CORIENNE study. *Hum Vaccin Immunother* 2016;**12**:1900–5. doi:10.1080/21645515.2016.1143577

2 Jan A, Hoek V, Andrews N, *et al.* The effect of underlying clinical conditions on the risk of developing invasive pneumococcal disease in England. *J Infect* 2012;**65**:17–24. doi:10.1016/j.jinf.2012.02.017

3 Puig-Barberà J, Natividad-Sancho A, Trushakova S, *et al.* Epidemiology of Hospital Admissions with Influenza during the 2013 / 2014 Northern Hemisphere Influenza Season: Results from the Global Influenza Hospital Surveillance Network. *PLoS One* 2016;**11**:e0154970. doi:10.1371/journal.pone.0154970

4 Kerkhove MD Van, Vandemaele KAH, Shinde V, *et al.* Risk Factors for Severe Outcomes following 2009 Influenza A (H1N1) Infection: A Global Pooled Analysis. *PLoS Med* 2011;**8**:e1001053. doi:10.1371/journal.pmed.1001053

5 Polakos NK, Cornejo JC, Murray DA, *et al.* Kupffer Cell-Dependent Hepatitis Occurs during Influenza Infection. *Am J Pathol* 2006;**168**:1169–78. doi:10.2353/ajpath.2006.050875

6 Duchini A, Viernes ME, Nyberg LM, *et al.* Hepatic Decompensation in Patients With Cirrhosis During Infection With Influenza A. *Arch Intern Med* 2000;**160**:113–5. doi:10.1001/archinte.160.1.113

7 World Health Organization. Weekly epidemiological record: Vaccines against influenza. WHO position paper. 2012;:461–76.http://www.who.int/wer/2012/wer8747/en/ (accessed 13 Jun 2017).

8 Fierer J, Finley F. Deficient Serum Bactericidal Activity Against Escherichia Coli in Patients with Cirrhosis of the Liver. *J Clin Invest* 1979;**63**:912–21. doi:10.1172/JCI109391

9 Hassner A, Kletter Y, Shlag D, *et al.* Impaired monocyte function in liver cirrhosis. *Br Med J (Clin Res Ed)* 1981;**282**:1262–3.

10 Brien AJO, Fullerton JN, Massey KA, *et al.* Prostaglandin E2 Mediates Immunosuppression in Acutely Decompensated Cirrhosis. *Nat Med* 2014;**20**:518–23. doi:10.1038/nm.3516

11 Rajkovic IA, Williams R. Abnormalities of Neutrophil Phagocytosis,

For peer review only

Intracellular Killing and Metabolic Activity in Alcoholic Cirrhosis and Hepatitis. *Hepatology* 1986;**6**:252–62. doi:10.1002/hep.1840060217

12 Kanitz EE, Wu LA, Giambi C, *et al.* Variation in adult vaccination policies across Europe: An overview from VENICE network on vaccine recommendations, funding and coverage. *Vaccine* 2012;**30**:5222–8. doi:10.1016/j.vaccine.2012.06.012

13 European Centre for Disease Prevention and Control. Seasonal influenza vaccination and antiviral use in Europe – Overview of vaccination recommendations and coverage rates in the EU Member States for the 2013–14 and 2014–15 influenza seasons. 2016.http://ecdc.europa.eu/en/publications/_layouts/forms/Publication_DispForm.aspx?List=4f55ad51-4aed-4d32-b960-af70113dbb90&ID=1528 (accessed 13 Jun 2017).

14 Pebody RG, Leino T, Nohynek H, *et al.* Pneumococcal vaccination policy in Europe. Eurosurveillance. 2005;**10**.http://www.eurosurveillance.org/ViewArticle.aspx?ArticleId=564 (accessed 13 Jun 2017).

15 Public Health England. Influenza immunisation programme for England: GP patient groups - Data collection survey Season 2015 to 2016. 2016.https://www.gov.uk/government/statistics/seasonal-flu-vaccine-uptake-in-gp-patients-in-england-winter-season-2015-to-2016 (accessed 13 Jun 2017).

16 Blachier M, Leleu H, Peck-Radosavljevic M, *et al.* The burden of liver disease in Europe: a review of available epidemiological data. *J Hepatol* 2013;**58**:593–608. doi:10.1016/j.jhep.2012.12.005

17 Petticrew M, Shekelle P, Stewart LA, *et al.* Preferred reporting items for systematic review and meta-analysis protocols (PRISMA-P) 2015: elaboration and explanation. *BMJ* 2015;**349**:g7647. doi:10.1136/bmj.g7647

18 Kastner M, Wilczynski NL, Walker-Dilks C, *et al.* Age-Specific Search Strategies for Medline. *J Med Internet Res* 2006;**8**:e25. doi:10.2196/jmir.8.4.e25

19 The Cochrane Collaboration. Cochrane Handbook for Systematic Reviews

For peer review only

of Interventions. 2011.www.handbook.cochrane.org (accessed 13 Jun 2017).

20 Chalmers JD, Campling J, Dicker A, *et al.* A systematic review of the burden of vaccine preventable pneumococcal disease in UK adults. *BMC Pulm Med* 2016;:1–11. doi:10.1186/s12890-016-0242-0

21 University of Texas School of Public Health. Search filters for case-control studies, cohort studies, cross-sectional studies, clinical trials. 2012.http://libguides.sph.uth.tmc.edu/search_filters/ovid_psycinfo_filters (accessed 13 Jun 2017).

22 Scottish Intercollegiate Guidelines Network. SIGN search filters for observational studies.

23 BMJ Evidence Centre. Medline cohort and case-control strategy. 2012.http://clinicalevidence.bmj.com/x/set/static/ebm/learn/665076.html (accessed 13 Jun 2017).

24 Moher D, Liberati A, Tetzlaff J, *et al.* Preferred Reporting Items for Systematic Reviews and Meta-Analyses: The PRISMA Statement. *Annu Intern Med* 2009;**151**:264–9. doi:10.1371/journal.pmed1000097

25 World Health Organization. International Clinical Trials Registry Platform Search Portal. http://apps.who.int/trialsearch/ (accessed 13 Jun 2017).

26 Kirkham JJ, Dwan KM, Altman DG, *et al.* The impact of outcome reporting bias in randomised controlled trials on a cohort of systematic reviews. *BMJ* 2010;**340**:c365. doi:10.1136/bmj.c365

27 Guyatt GH, Oxman AD, Vist GE, *et al.* GRADE: an emerging consensus on rating quality of evidence and strength of recommendations. *BMJ* 2008;**336**:924–6. doi:10.1136/bmj.39489.470347.AD

For peer review only

PRISMA-P (Preferred Reporting Items for Systematic review and Meta-Analysis Protocols) 2015 checklist: recommended items to address in a systematic review protocol*

Section and topic	Item No	Checklist item	Status	Page
**ADMINISTRATIVE INFORMATION**				
Title:				
Identification	1a	Identify the report as a protocol of a systematic review	Completed	1
Update	1b	If the protocol is for an update of a previous systematic review, identify as such	n/a	n/a
Registration	2	If registered, provide the name of the registry (such as PROSPERO) and registration number	Completed	2, 21
Authors:				
Contact	3a	Provide name, institutional affiliation, e-mail address of all protocol authors; provide physical mailing address of corresponding author	Completed	1
Contributions	3b	Describe contributions of protocol authors and identify the guarantor of the review	Completed	21
Amendments	4	If the protocol represents an amendment of a previously completed or published protocol, identify as such and list changes; otherwise, state plan for documenting important protocol amendments	Completed	17
Support:				
Sources	5a	Indicate sources of financial or other support for the review	Completed	21
Sponsor	5b	Provide name for the review funder and/or sponsor	Completed	21
Role of sponsor or funder	5c	Describe roles of funder(s), sponsor(s), and/or institution(s), if any, in developing the protocol	Completed	21
**INTRODUCTION**				
Rationale	6	Describe the rationale for the review in the	Completed	4-6

For peer review only

		context of what is already known		
Objectives	7	Provide an explicit statement of the question(s) the review will address with reference to participants, interventions, comparators, and outcomes (PICO)	Completed	7
**METHODS**				
Eligibility criteria	8	Specify the study characteristics (such as PICO, study design, setting, time frame) and report characteristics (such as years considered, language, publication status) to be used as criteria for eligibility for the review	Completed	7-9
Information sources	9	Describe all intended information sources (such as electronic databases, contact with study authors, trial registers or other grey literature sources) with planned dates of coverage	Completed	9
Search strategy	10	Present draft of search strategy to be used for at least one electronic database, including planned limits, such that it could be repeated	Completed	9-12
Study records:				
Data management	11a	Describe the mechanism(s) that will be used to manage records and data throughout the review	Completed	13
Selection process	11b	State the process that will be used for selecting studies (such as two independent reviewers) through each phase of the review (that is, screening, eligibility and inclusion in meta-analysis)	Completed	13
Data collection process	11c	Describe planned method of extracting data from reports (such as piloting forms, done independently, in duplicate), any processes for obtaining and confirming data from investigators	Completed	13-14
Data items	12	List and define all variables for which data will be sought (such as PICO items, funding sources), any pre-planned data assumptions and simplifications	Completed	14-15
Outcomes and	13	List and define all outcomes for which data will	Completed	15-16

For peer review only

prioritization		be sought, including prioritization of main and additional outcomes, with rationale		
Risk of bias in individual studies	14	Describe anticipated methods for assessing risk of bias of individual studies, including whether this will be done at the outcome or study level, or both; state how this information will be used in data synthesis	Completed	16-17
Data synthesis	15a	Describe criteria under which study data will be quantitatively synthesised	Completed	17
	15b	If data are appropriate for quantitative synthesis, describe planned summary measures, methods of handling data and methods of combining data from studies, including any planned exploration of consistency (such as I^2, Kendall's τ)	Completed	17-19
	15c	Describe any proposed additional analyses (such as sensitivity or subgroup analyses, meta-regression)	Completed	19
	15d	If quantitative synthesis is not appropriate, describe the type of summary planned	Completed	19-20
Meta-bias(es)	16	Specify any planned assessment of meta-bias(es) (such as publication bias across studies, selective reporting within studies)	Completed	20
Confidence in cumulative evidence	17	Describe how the strength of the body of evidence will be assessed (such as GRADE)	Completed	20

*** It is strongly recommended that this checklist be read in conjunction with the PRISMA-P Explanation and Elaboration (cite when available) for important clarification on the items. Amendments to a review protocol should be tracked and dated. The copyright for PRISMA-P (including checklist) is held by the PRISMA-P Group and is distributed under a Creative Commons Attribution Licence 4.0.**

From: Shamseer L, Moher D, Clarke M, Ghersi D, Liberati A, Petticrew M, Shekelle P, Stewart L, PRISMA-P Group. Preferred reporting items for systematic review and meta-analysis protocols (PRISMA-P) 2015: elaboration and explanation. BMJ. 2015 Jan 2;349(jan02 1):g7647.

For peer review only

BMJ Open

EFFECTIVENESS OF PNEUMOCOCCAL AND INFLUENZA VACCINES TO PREVENT SERIOUS HEALTH COMPLICATIONS IN ADULTS WITH CHRONIC LIVER DISEASE: A PROTOCOL FOR A SYSTEMATIC REVIEW

Journal:	*BMJ Open*
Manuscript ID	bmjopen-2017-018223.R2
Article Type:	Protocol
Date Submitted by the Author:	31-Jan-2018
Complete List of Authors:	Härmälä, Suvi; University College London, Institute of Health Informatics<br>Parisinos, Constantinos; University College London, Institute of Health Informatics<br>Shallcross, Laura; University College London, Institute of Health Informatics<br>O'Brien, Alastair; University College London, Division of Medicine<br>Hayward, Andrew; University College London, Institute of Epidemiology and Health Care
**Primary Subject Heading**:	Infectious diseases
Secondary Subject Heading:	Gastroenterology and hepatology, Public health
Keywords:	Pneumococcal vaccine, Influenza vaccine, Chronic liver disease

For peer review only

SCHOLARONE™ Manuscripts

EFFECTIVENESS OF PNEUMOCOCCAL AND INFLUENZA VACCINES TO PREVENT SERIOUS HEALTH COMPLICATIONS IN ADULTS WITH CHRONIC LIVER DISEASE: A PROTOCOL FOR A SYSTEMATIC REVIEW

Suvi Härmälä[1], Constantinos Parisinos[1], Laura Shallcross[1], Alastair O'Brien[2] & Andrew Hayward[1,3]

[1] Institute of Health Informatics, University College London, London, United Kingdom.

[2] Division of Medicine, University College London, London, United Kingdom.

[3] Institute of Epidemiology and Health Care, University College London, London, United Kingdom.

Corresponding author: Suvi K. Härmälä, Institute of Health Informatics, University College London, 222 Euston Road, London NW1 2DA, United Kingdom, suvi.harmala.14@ucl.ac.uk, +44 (0)20 3549 5338

Keywords: Pneumococcal vaccine, influenza vaccine, chronic liver disease

Word count (excluding title page, abstract, strengths & limitations, references and table): 3909

For peer review only

ABSTRACT

Introduction: In advanced chronic liver disease, diseases caused by common bacteria *Streptococcus pneumoniae* or influenza virus put people at an increased risk of serious health complications and death. The effectiveness of the available vaccines in reducing the risk of poor health outcomes, however, is less clear.

Methods and analysis: We will search MEDLINE (Ovid), EMBASE (Ovid), Pubmed and Cochrane Central Register of Controlled Trials (CENTRAL) for published reports on randomised controlled trials and observational studies on the effectiveness of pneumococcal and influenza vaccines in people with chronic liver disease. Two independent reviewers will screen the studies for eligibility, extract data and assess study quality and risk of bias. Random effects meta-analyses will be performed as appropriate.

Ethics and dissemination: Formal ethical approval is not required, as no primary data will be collected for this study. We will publish results of this study in relevant peer-reviewed medical journal or journals. Where possible, the study results will also be presented as posters or talks at relevant medical conferences and meetings.

Prospero registration number: CRD42017067277

For peer review only

STRENGTHS AND LIMITATIONS OF THIS STUDY:

- This study protocol follows the recommendations by the Preferred Reporting Items for Systematic Review and Meta-Analysis Protocols (PRISMA-P).
- This study protocol has been prospectively registered in the International Prospective Register of Systematic Reviews (PROSPERO).
- Our comprehensive search strategy will minimise the risk of missing relevant studies, in particular, those with a randomised design.
- The selection of studies, data extraction, the risk of bias and quality of evidence assessments will be conducted by two independent authors.
- Inclusion of studies with a non-randomised design may decrease the overall quality of the body of evidence for the study outcomes.

For peer review only

BACKGROUND

Burden of pneumococcal and influenza infections in chronic liver disease

In advanced chronic liver disease, as the immune function progressively deteriorates, diseases caused by common bacteria *Streptococcus pneumoniae* or influenza virus can lead to serious health complications and death.

In Spain in 2011, the population-level annual incidence rate for pneumococcal pneumonia-related hospitalisation in adults with liver disease was estimated at approximately 540 per 100 000 compared to approximately 6 per 100 000 without at-risk conditions,[1]. Adults with liver disease were over 50 times more likely to be hospitalised for pneumococcal pneumonia than adults without at-risk conditions,[1]. Similarly, in England in 2008/2009, for invasive pneumococcal disease (IPD), such as meningitis, bacteraemia and sepsis, the annual incidence of hospitalisation in adults with liver disease was estimated at about 100 per 100 000 compared to about 8 per 100 000 in the healthy population,[2]. Approximately 37% of liver disease patients hospitalised for IPD died, compared to 5% of patients without underlying risk conditions,[2]. Chronic liver disease patients were over 30 times more likely to be admitted to hospital and 10 times more likely to die during the IPD-related hospitalisation than adults without at-risk conditions,[2].

Although there are no population-level estimates of severe influenza incidence in people with chronic liver disease, evidence suggests that liver disease patients are at an increased risk of health complications from influenza. A 2-fold increased risk of influenza admission was observed in liver disease patients at 19 hospitals in Russia, Turkey, China, and Spain during the 2013/2014 season,[3]. Similarly, an analysis of data on laboratory-confirmed influenza cases collected from several World Health Organisation (WHO) member states during the 2009 influenza A (H1N1) pandemic found liver disease patients to have a greater than 5-fold increased risk of influenza-related hospitalisation and over 17-fold increased risk of death compared to that of healthy individuals,[4].

For peer review only - http://bmjopen.bmj.com/site/about/guidelines.xhtml

Furthermore, influenza infection, while not directly targeting the liver, may cause collateral transient liver damage,[5] and trigger hepatic decompensation (such as ascites and hepatic encephalopathy) in liver disease patients,[6].

Types of pneumococcal and influenza vaccines, vaccination policy and vaccine uptake

Two types of vaccines, polysaccharide pneumococcal vaccines including serotypes of *S. pneumoniae* (PPV23) and conjugate pneumococcal vaccines including 7 (PCV7), 10 (PCV10) or 13 (PCV13) *S. pneumoniae* serotypes, are available to protect against pneumococcal infection. None of these contains live bacteria.The most commonly used influenza vaccines are injectable, inactivated vaccines that contain either inactivated whole influenza virus or split or subunit virus products. These vaccines protect either against two influenza A (H1N1 and H3N2) strains and one influenza B strain (trivalent vaccines) or two influenza A and two influenza B strains (quadrivalent vaccines). New vaccines are developed every year to protect against the prevailing strains of the upcoming influenza season and a yearly vaccination is recommended to ensure continued protection. Live attenuated influenza vaccines exist, however, these may not be suitable for people with chronic co-morbidities,[7]. Immune response defects associated with advanced liver disease, [8–11] may also dampen the response to vaccines.

The majority of European countries recommend both adult influenza (29/29 countries) and pneumococcal vaccination (22/29 countries) for specific high-risk groups,[12]. Chronic liver disease patients, however, may not be included in these high-risk target groups in all countries. Whilst 90% (27/ 30) of the countries recommended influenza immunisation for people with liver disease during the 2014-2015 influenza season,[13], only 43% (6/14) of countries surveyed in 2005 recommended pneumococcal vaccination for chronic liver disease patients,[14]. Moreover, whilst the adult vaccination recommendations for influenza are supported by official funding mechanisms in most countries (21/29), the cost of the pneumococcal vaccination is covered by only half (11/22),[12].

For peer review only - http://bmjopen.bmj.com/site/about/guidelines.xhtml

Uptake of influenza vaccine in people with chronic diseases in general is poor. The median coverage rate across Europe in the 2014/2015 season was less than 50% (only 7/30 countries were able to provide separate coverage data for individuals with chronic medical conditions),[13]. The situation in liver disease patients as a separate group seems no different with less than 50% of working-age liver disease patients in the UK covered by the influenza vaccine in 2015/2016 season,[15]. Although fewer data exist concerning the uptake of pneumococcal adult vaccination,[12], its uptake in liver disease patients is not likely to be any higher than that of influenza vaccine.

Rationale for the review

Approximately 29 million people in Europe alone are affected by some form of liver disease,[16]. Worryingly, due to the increasing prevalence of obesity and persistence of other liver damage risk factors such as alcohol abuse and hepatitis infections, this number is expected to grow,[16]. Whilst evidence suggests that following infection with influenza or *S. pneumoniae*, people with liver disease have a higher than average risk of poor health outcomes, the effectiveness of the vaccines in reducing this risk is less clear and warrants further investigation.

To our knowledge, no systematic review to date has investigated the effects of pneumococcal and influenza vaccines in preventing poor health outcomes in chronic liver disease. The present review intends to fill this gap by providing a systematic synthesis of the available evidence. The results of this review may inform future vaccination strategies and help improve vaccination coverage. This, in turn, may have a positive impact on both the number of influenza and pneumococcal disease-related hospital admissions and patient outcomes in liver disease.

OBJECTIVES

For peer review only - http://bmjopen.bmj.com/site/about/guidelines.xhtml

The aim of this review is to assess the effectiveness of pneumococcal and influenza vaccines to prevent serious health complications in adults with chronic liver disease.

The objectives are:

- To assess the effectiveness of pneumococcal and influenza vaccines to prevent hospitalisation in adults with chronic liver disease.
- To assess the effectiveness of pneumococcal and influenza vaccines to prevent death in adults with chronic liver disease.
- To assess the effects of pneumococcal and influenza vaccines for eliciting a serological response in adults with chronic liver disease.

For peer review only

METHODS

This study protocol follows the recommendations by the Preferred Reporting Items for Systematic Review and Meta-Analysis Protocols (PRISMA-P) 2015,[17].

Eligibility criteria

Types of studies

We will include all randomized clinical trials, cohort (with comparison group/s) and case-control studies that investigate the effectiveness of pneumococcal or influenza vaccines for preventing hospitalisation or death in adults with chronic liver disease. We will also include all randomized clinical trials, cohort (with or without comparison group/s) and case-control studies that report the serological response to one or both of these vaccines in adults with chronic liver disease. We will only include published studies in English language and studies that have been published or accepted for publication. We will exclude review articles, case reports, cross-sectional studies, animal studies, editorials, clinical guidelines and any studies that have been fully or partially retracted from publication. Patients included in multiple studies will be reported only once.

Types of participants

We will include studies that enrol 18+ year-old adult patients with chronic liver disease of any severity (non-cirrhotic, cirrhotic) or aetiology (viral, alcoholic, non-alcoholic fatty liver, autoimmune).

Types of interventions

We will include studies that investigate the effects of a conjugate or polysaccharide pneumococcal vaccine (against *S. pneumoniae*) and/or an inactivated (whole virus, split virus or subunit), injectable influenza vaccine. Vaccines can be adjuvanted, intradermal and of any dose. We will exclude live, recombinant, virosomal and experimental vaccines.

Types of comparators

We will include studies comparing one or both of the vaccines of interest to a placebo, an alternative intervention or no intervention. We will also include studies without a comparison group when the outcome studied is the serological response to the vaccine.

Types of outcome measures

We will include studies that report on one or more of our primary outcomes and/or our secondary outcome of interest.

Primary outcomes

- All-cause hospitalisation
- All-cause mortality

Secondary outcomes

- Serological response to vaccine

For peer review only

- Acute respiratory illness-related hospitalisation
- Influenza illness or influenza-like-illness (ILI)-related hospitalisation (based on hospital discharge codes or clinical diagnosis)
- Pneumococcal disease-related hospitalisation
- Hospitalisation for liver disease complications (variceal bleeding, hepatic encephalopathy, ascites, spontaneous bacterial peritonitis, jaundice and bacteraemia or sepsis)
- Acute respiratory illness-related mortality
- Influenza illness or ILI-related mortality
- Pneumococcal disease-related mortality
- Liver disease-related mortality

Information sources

Electronic searches

To capture all relevant studies, we plan to search the following databases:

- Cochrane Central Register of Controlled Trials (CENTRAL)
- MEDLINE (Ovid)
- EMBASE (Ovid)
- PubMed
-

Each database will be searched separately and the search strategy first developed in MEDLINE will be adapted to each database interface as appropriate. We plan to also search relevant studies from the reference lists of the eligible studies identified through the electronic searches.

Search strategy

We will use two complementary strategies to identify relevant articles. First, we will identify articles reporting outcomes of vaccination in patients with liver disease by combining search terms for influenza and pneumococcal vaccination

For peer review only

with search terms for chronic liver disease (Search 1). This search will be filtered by study design. The provisional search terms for liver disease and pneumococcal and influenza vaccines are listed in Table 1.
Recognising that liver disease patients may be included as subgroups in clinical trials of vaccination, we will also search for randomised controlled trials of influenza and pneumococcal vaccine that have recruited individuals from the general population (Search 2).

To search for studies with adult participants, we will combine the geriatric and adult medicine-specific search strategies by Kastner et al.,[18]. In order to maximise the sensitivity of the searches for randomised clinical trials, we will use a filter that combines terms from the Cochrane Highly Sensitive Search Strategy,[19] and less specific version of the same filter by Chalmers et al.,[20] to identify randomised trials. Similarly, to maximise the sensitivity of searches for case-control and cohort studies, we will use a filter that combines terms from the University of Texas School of Public Health filter for observational studies,[21], SIGN observational study filter,[22] and the BMJ Evidence Centre case-control and cohort strategy,[23]. We will limit our search to studies including adult human study subjects but not based on the setting/country or publication year of the articles. The search terms for adult and study design filters are listed in Table 1.

Table 1. MEDLINE (Ovid) provisional search terms

Search concept	Search terms
Pneumococcal vaccine	1. exp Pneumococcal Vaccines/
	2. exp Pneumococcal Infections/pc [Prevention & Control]
	3. ((anti?pneum* or pneum*) adj5 (vaccin* or immuni*)).mp.
	4. (PPV?23* or PPSV or PPSV?23* or PCV?7* or PCV?10* or PCV?13*).mp.
	5. ((PPV or PCV) adj5 (pneum* or vaccin* or immuni*)).mp.
	6. ((7?valent or hepta?valent or 10?valent or 13?valent or

For peer review only

	23?valent) adj5 (vaccin* or immuni*)).mp.
	7. 1 or 2 or 3 or 4 or 5 or 6 or 7
Influenza vaccine	1. Influenza Vaccines/
	2. Influenza, Human/pc [Prevention & Control]
	3. ((anti?influenza or influenza or seasonal or anti?flu or flu) adj5 (vaccin* or immuni*)).mp.
	4. ((TIV or QIV or trivalent or quadrivalent or 3?valent or 4?valent) adj5 (vaccin* or immuni*)).mp.
	5. 1 or 2 or 3 or 4 or 5
Liver disease	1. exp Liver Diseases/
	2. ((liver or hepat*) adj3 disease*).mp.
	3. ("chronic liver" or "chronic hepat*").mp.
	4. cirrho*.mp.
	5. 1 or 2 or 3 or 4
Adult participants	1. exp Adult/
	2. adult.mp.
	3. (middle?aged or aged).sh.
	4. age*.tw.
	5. 1 or 2 or 3 or 4
Randomised controlled trials	1. randomized controlled trial.pt.
	2. randomi*.ab,ti.
	3. randomly.ab,ti.
	4. controlled clinical trial.pt.
	5. trial.ab,ti.
	6. groups.ab,ti.
	7. drug therapy.fs.
	8. placebo.ab,ti.
	9. 1 or 2 or3 or 4 or 5 or 6 or 7 or 8
	10. Animals/

For peer review only - http://bmjopen.bmj.com/site/about/guidelines.xhtml

	11. Humans/
	12. 10 not (10 and 11)
	13. 9 not 12
Case-control and cohort studies	1. Epidemiologic Studies/
	2. exp Case control studies/
	3. exp Cohort studies/
	4. Longitudinal studies/
	5. Follow up studies/
	6. Prospective studies/
	7. Retrospective studies/
	8. Control groups/
	9. Matched-Pair Analysis/
	10. (Case* adj5 control*).ti,ab,kw.
	11. (Case* adj5 comparison*).ti,ab,kw.
	12. Control group*.ti,ab,kw.
	13. (Cohort adj (study or studies)).ti,ab.
	14. Cohort anal*.ti,ab.
	15. (Follow up adj (study or studies)).ti,ab.
	16. (Observational adj (study or studies)).ti,ab.
	17. Longitudinal.ti,ab.
	18. Retrospective.ti,ab.
	19. Prospective.ti,ab.
	20. 1 or 2 or 3 or 4 or 5 or 6 or 7 or 8 or 9 or 10 or 11 or 12 or 13 or 14 or 15 or 16 or 17 or 18 or 19
	21. Animals/
	22. Humans/
	23. 21 not (21 and 22)
	24. 20 not 23

Study records

For peer review only

Data management

The search results will be uploaded into reference management software (Mendeley) to remove duplicate records of the same report. The unique records will then be uploaded into web-based, systematic review management software (DistillerSR). Both the initial abstract and title screening and the full-text review and extraction of data from the eligible studies will be performed using standardised, pre-created online forms. All forms will be piloted and revised as needed by the reviewers before starting the review.

Selection process

Articles that have been identified through the broad search of RCTs of pneumococcal or flu vaccination in the general population (Search 2) will first be pre-screened by title by one reviewer (SH). Articles meeting pre-screening criteria will be combined with the articles identified through Search 1. These articles will then all be screened by two independent reviewers (SH&CP) by abstract and title. Where the study eligibility cannot be established based on the title and abstract, the report will be passed on to the full-text review. Similarly, records subject to disagreement over eligibility will be included in the full-text review.

The full-text review will be independently completed for all eligible articles by two independent reviewers authors (SH&CP). Reasons for exclusion of ineligible studies will be recorded. Disagreements will be resolved by consulting a third review author (AO) and any uncertainties by correspondence with study investigators. Multiple reports of the same study will be collated into one and, where not possible, only the most relevant report based on our eligibility criteria will be included. The study selection process will be recorded and presented in flow diagram format according to the recommendations of Preferred Reporting Items for Systematic Reviews and Meta-Analyses (PRISMA),[24].

For peer review only

Data collection process

The data will be extracted and entered into standardised, pre-created online data extraction forms independently and in duplicate by two review authors (SH&CP). Disagreement will be resolved by consulting a third review author (LS) and uncertainties by correspondence with study investigators.

Data items

We will extract data on:

- Study participants: inclusion and exclusion criteria, method of recruitment/selection, study population characteristics and any imbalances at baseline (sex, age, aetiology and severity of liver disease, co-morbidities, alcohol use, smoking status, pre-vaccination infection status, medication/treatment other than intervention).
- Interventions and comparators (vaccine type, comparison treatment, dose, route of delivery, number and timing of vaccinations/comparator treatments, number of individuals in intervention and comparison group, follow-up time in intervention and comparison groups)
- Outcomes (definition, time points measured and reported, unit of measurement, number of outcomes in the intervention and control group, unadjusted and adjusted effect measures, covariates that the effect measures were adjusted for, comparisons, missing data and reasons for missingness, statistical methods used, processes for randomisation e.g. allocation concealment)
- Study designs and methods (study type, country and setting, date of study, study duration, aim of study, withdrawals).
- Study quality and study bias (according to the needs of the assessments specified below).
- Study funding and conflicts of interest

Effect measures will be collected in the format in which they are reported and transformed for presentation and analysis if appropriate.

For peer review only - http://bmjopen.bmj.com/site/about/guidelines.xhtml

Outcomes and prioritisation

Our main outcomes of interest are hospitalisation and death. These are potential severe outcomes of influenza illness and pneumococcal disease. It may be challenging to identify and establish, especially if hospital discharge records are reviewed retrospectively, the exact cause of the hospitalisation or death of a patient with an underlying chronic condition. For this reason, our primary outcomes we will include all causes. Additionally, studies may not always specify whether a patient with a diagnosis of an infectious disease or liver disease complication was actually hospitalised. Unless it is specified that the patient was hospitalised or the illness was recorded in the hospital records, we will assume the patient was not hospitalised.

Our secondary outcomes of interest are the serological response to pneumococcal and influenza vaccines and a range of cause-specific hospitalisation and mortality. Serological response to a vaccine is an indicator of the vaccine's effect on building protective immunity against the disease the vaccination targets. Since, however, it is difficult to know what level of antibody or increase in antibody concentration may provide protection in people with chronic liver disease, we will evaluate both the post-vaccination antibody level and the pre- to post-vaccination fold change in geometric mean antibody concentrations. We will include studies where blood was drawn both before vaccination and at least 2 weeks after vaccination. This effect will be evaluated both for short-term (<6 months) and long-term (≥6 months) after vaccination. In case antibody responses are reported at multiple short-term or long-term time points, we will consider the time point closest to the timing of vaccination as the as the short-term response and the time point closest to 6 months as the long-term response. The more detailed causes of hospitalisation and death, allow us to understand about the more specific effects of the vaccines.

Assessment of risk of bias in individual studies

For peer review only - http://bmjopen.bmj.com/site/about/guidelines.xhtml

We will use the Cochrane Collaborations tool,[19] for assessing the risk of bias in all studies included in the review after the full-text review. The level of risk of bias in random sequence generation, allocation concealment, blinding of participants and personnel, blinding of outcome assessment, incomplete outcome data, selective reporting and other sources will be judged as “low”, “high” or “unclear” according to the criteria specified in the Cochrane Handbook,[19].

In the non-randomised studies, we will additionally assess the level of risk of confounding bias due to inadequately addressed differences between groups (i.e. is the effect estimate likely to be biased due to unaccounted confounding). We will consider age, sex, severity and aetiology of liver disease to be the most important potential confounders. We will judge the risk of confounding bias to be low if the study addressed the presence of these confounders by restricting participant selection by confounders, demonstrating balance between groups, matching on the confounders or adjusting for the confounders in statistical analyses of the effect size. The risk of confounding bias will be judged low if the confounders were adjusted for, high if the presence of the confounders was not addressed and unclear if there was insufficient information to judge the level of risk.

Two review authors (SH&CP) will independently assess the studies for each of the risk areas by entering a quote from the study to describe the procedures, their judgement together with a justification of the judgement into pre-created online forms in DistillerSR. Disagreements will be resolved by consulting a third review author (AH). The risk of bias assessments will be presented in a figure that shows the level of risk in the different risk areas within each individual study and in a graph that describes the proportion of studies within each risk level per risk area.

Assessment of bias in conducting the systematic review

For peer review only - http://bmjopen.bmj.com/site/about/guidelines.xhtml

We will conduct the systematic review following this pre-specified protocol and report any differences between the methods of the complete review and this protocol in the review.

Data synthesis

Criteria for quantitative data synthesis

We plan to carry out a formal meta-analysis only where more than a single study per outcome is identified and the study designs, protocols and measures of treatment effect are considered similar enough to produce a meaningful pooled effect.

Measures of treatment effect

For dichotomous data, the treatment effect will be estimated and presented as a risk ratio with 95% confidence intervals. For time-to-event data, we will present the results as a log hazard ratio with its standard error. For studies reporting on serological response using a cohort design without a comparison group, we will present the effect of vaccination as the post-vaccination antibody level and the pre- to post-vaccination fold change in geometric mean antibody concentration.

Unit of analysis issues

The outcomes will be analysed at the level of study participants from each individual study.

Dealing with missing data

We will contact investigators to obtain numerical outcome data that have not been fully reported (for instance where when a study is identified as an abstract only or outcomes are reported in figures only). Where possible, we will calculate missing standard deviations from other reported statistics such as confidence

For peer review only

intervals or standard errors. The impact of including studies with high levels of missing outcome data on the treatment effect will be explored in the sensitivity analysis.

Assessment of heterogeneity

To assess heterogeneity between studies, we plan to present a forest plot for each of the review outcomes. We will then calculate the formal heterogeneity variance statistics τ^2, I^2 and the Q-statistic. We will regard heterogeneity as substantial if τ^2 is greater than 0, I^2 is more than 30% and the p-value for Q-statistic is less than 0.10. We plan to further explore the potential causes of substantial heterogeneity in the subgroup analyses or meta-regression (specified below).

Quantitative data synthesis

Statistical analyses will be performed using Stata or R Studio. To account for the presence of heterogeneity, we will use random-effects meta-analysis to summarise the average effects of vaccination on the defined outcomes across studies. The results will be presented in forest plots with the average treatment effect (RR) with 95% confidence intervals, and the estimates of τ^2 and I^2. We will use the pooled average treatment effect to calculate the effectiveness of the vaccines (100*[1-RR]) in preventing the primary outcomes. The immunogenetic effect of the vaccines will be summarised as the mean post-vaccination antibody level with 95% confidence intervals and the mean pre- to post-vaccination fold change in geometric mean antibody concentration with 95% confidence intervals. Observational studies and randomised controlled trial studies will be considered in separate analyses.

Subgroup analysis and investigation of heterogeneity

In case we identify an adequate number of studies (studies per explanatory variable ≥10) we plan to investigate the potential causes of heterogeneity

For peer review only - http://bmjopen.bmj.com/site/about/guidelines.xhtml

between studies through random effects meta-regression analyses. We will consider the following categories of explanatory variables: severity of liver disease, aetiology of liver disease and the reason for hospital admission/ mortality (primary outcomes). Inclusion/exclusion of the explanatory variables in the heterogeneity investigations will depend on the characteristics and design of the identified studies. If we do not identify enough studies to perform meta-regressions but there are a minimum 5 studies per analysis we plan to carry out subgroup analyses to investigate whether these explanatory variables can explain heterogeneity between the studies.

Sensitivity analysis

In case the identified studies differ in terms of risk of bias, we plan to investigate the impact of excluding studies with high/unclear risk of bias on effect estimates in sensitivity analyses.

Qualitative data synthesis

We will provide a narrative summary of the study results for all outcomes, categorised by study design and vaccine type (influenza vaccine and pneumococcal vaccine). For the primary outcomes, we will report the cause of hospitalisation and death studied. Characteristics (participants, interventions, comparators, outcomes, study design and methods and notes on funding and conflicts of interests) of all studies included in the review will also be presented in separate tables. The results for outcomes where meta-analysis will not be carried out due to insufficient homogeneity between studies will be presented in forest plots without the pooled effect estimate.

Meta-bias(es)

Assessment of reporting biases across studies

For peer review only

We plan to investigate reporting bias using funnel plots. If there are enough studies in the analysis (minimum 10), we will also carry out the Egger's test to assess whether there is a linear association between the study's result and its standard error.

We plan to assess selective outcome reporting bias by comparing what the study set to measure and analyse in the methods section of the study report (for studies published after 2006, we will also investigate the details trial protocol if it can be identified through the WHO International Clinical Trials Registry Platform,[25] launched in 2007) with the results that were reported. Using the Outcome Reporting Bias in Trials (ORBIT) classification system,[26] we will evaluate whether the risk of selective outcome reporting bias is present and whether the risk is low or high.

Confidence in cumulative evidence

We will use the Grading of Recommendations Assessment, Development and Evaluation (GRADE) Working Group system,[27] to assess and report the overall quality of the body of evidence for each outcome studied. The within-study risk of bias (methodological quality), directness of evidence, heterogeneity, the precision of effect estimates and risk of publication bias will be independently assessed by two review authors (SH&CP). The quality of evidence will be judged and reported as "high", "moderate", "low", or "very low" following the Cochrane Handbook for Systematic Reviews of Interventions guidelines,[19].

COMPETING INTERESTS

The authors declare no competing interests.

FUNDING

For peer review only - http://bmjopen.bmj.com/site/about/guidelines.xhtml

This work was supported by the UK Biotechnology and Biological Sciences Research Council grant number BBSRC BB/M009513/1 to SH.

AUTHOR CONTRIBUTIONS

The study was conceived by SH, LS, AO and AH. SH developed the eligibility criteria, search strategy, risk of bias assessment strategy and data extraction plan with guidance from LS, AO and AH. SH wrote the manuscript, to which all authors SH, CP, LS, AO and AH contributed.

ETHICS AND DISSEMINATION

Formal ethical approval is not required for this study, as no primary data will be collected. We will publish results of this study in relevant peer-reviewed medical journal or journals. Where possible, the study results will also be presented as posters or talks at relevant medical conferences and meetings.

PROTOCOL REGISTRATION

This systematic review protocol was registered in the International Prospective Register of Systematic Reviews (PROSPERO) on 13th June 2017 (registration number CRD42017067277).

REFERENCES

1 Gil-Prieto R, Pascual-Garcia R, Walter S, *et al.* Risk of hospitalization due to pneumococcal disease in adults in Spain. The CORIENNE study. *Hum Vaccin Immunother* 2016;**12**:1900–5. doi:10.1080/21645515.2016.1143577

2 Jan A, Hoek V, Andrews N, *et al.* The effect of underlying clinical conditions

For peer review only

on the risk of developing invasive pneumococcal disease in England. *J Infect* 2012;**65**:17–24. doi:10.1016/j.jinf.2012.02.017

3 Puig-Barberà J, Natividad-Sancho A, Trushakova S, *et al.* Epidemiology of Hospital Admissions with Influenza during the 2013 / 2014 Northern Hemisphere Influenza Season: Results from the Global Influenza Hospital Surveillance Network. *PLoS One* 2016;**11**:e0154970. doi:10.1371/journal.pone.0154970

4 Kerkhove MD Van, Vandemaele KAH, Shinde V, *et al.* Risk Factors for Severe Outcomes following 2009 Influenza A (H1N1) Infection: A Global Pooled Analysis. *PLoS Med* 2011;**8**:e1001053. doi:10.1371/journal.pmed.1001053

5 Polakos NK, Cornejo JC, Murray DA, *et al.* Kupffer Cell-Dependent Hepatitis Occurs during Influenza Infection. *Am J Pathol* 2006;**168**:1169–78. doi:10.2353/ajpath.2006.050875

6 Duchini A, Viernes ME, Nyberg LM, *et al.* Hepatic Decompensation in Patients With Cirrhosis During Infection With Influenza A. *Arch Intern Med* 2000;**160**:113–5. doi:10.1001/archinte.160.1.113

7 World Health Organization. Weekly epidemiological record: Vaccines against influenza. WHO position paper. 2012;:461–76.http://www.who.int/wer/2012/wer8747/en/ (accessed 13 Jun 2017).

8 Fierer J, Finley F. Deficient Serum Bactericidal Activity Against Escherichia Coli in Patients with Cirrhosis of the Liver. *J Clin Invest* 1979;**63**:912–21. doi:10.1172/JCI109391

9 Hassner A, Kletter Y, Shlag D, *et al.* Impaired monocyte function in liver cirrhosis. *Br Med J (Clin Res Ed)* 1981;**282**:1262–3.

10 Brien AJO, Fullerton JN, Massey KA, *et al.* Prostaglandin E2 Mediates Immunosuppression in Acutely Decompensated Cirrhosis. *Nat Med* 2014;**20**:518–23. doi:10.1038/nm.3516

11 Rajkovic IA, Williams R. Abnormalities of Neutrophil Phagocytosis, Intracellular Killing and Metabolic Activity in Alcoholic Cirrhosis and Hepatitis. *Hepatology* 1986;**6**:252–62. doi:10.1002/hep.1840060217

12 Kanitz EE, Wu LA, Giambi C, *et al.* Variation in adult vaccination policies across Europe: An overview from VENICE network on vaccine

For peer review only

recommendations, funding and coverage. *Vaccine* 2012;**30**:5222–8. doi:10.1016/j.vaccine.2012.06.012

13 European Centre for Disease Prevention and Control. Seasonal influenza vaccination and antiviral use in Europe – Overview of vaccination recommendations and coverage rates in the EU Member States for the 2013–14 and 2014–15 influenza seasons. 2016.http://ecdc.europa.eu/en/publications/_layouts/forms/Publication_DispForm.aspx?List=4f55ad51-4aed-4d32-b960-af70113dbb90&ID=1528 (accessed 13 Jun 2017).

14 Pebody RG, Leino T, Nohynek H, *et al.* Pneumococcal vaccination policy in Europe. Eurosurveillance. 2005;**10**.http://www.eurosurveillance.org/ViewArticle.aspx?ArticleId=564 (accessed 13 Jun 2017).

15 Public Health England. Influenza immunisation programme for England: GP patient groups - Data collection survey Season 2015 to 2016. 2016.https://www.gov.uk/government/statistics/seasonal-flu-vaccine-uptake-in-gp-patients-in-england-winter-season-2015-to-2016 (accessed 13 Jun 2017).

16 Blachier M, Leleu H, Peck-Radosavljevic M, *et al.* The burden of liver disease in Europe: a review of available epidemiological data. *J Hepatol* 2013;**58**:593–608. doi:10.1016/j.jhep.2012.12.005

17 Petticrew M, Shekelle P, Stewart LA, *et al.* Preferred reporting items for systematic review and meta-analysis protocols (PRISMA-P) 2015: elaboration and explanation. *BMJ* 2015;**349**:g7647. doi:10.1136/bmj.g7647

18 Kastner M, Wilczynski NL, Walker-Dilks C, *et al.* Age-Specific Search Strategies for Medline. *J Med Internet Res* 2006;**8**:e25. doi:10.2196/jmir.8.4.e25

19 The Cochrane Collaboration. Cochrane Handbook for Systematic Reviews of Interventions. 2011.www.handbook.cochrane.org (accessed 13 Jun 2017).

20 Chalmers JD, Campling J, Dicker A, *et al.* A systematic review of the burden of vaccine preventable pneumococcal disease in UK adults. *BMC Pulm Med*

For peer review only

For peer review only - http://bmjopen.bmj.com/site/about/guidelines.xhtml

2016;:1–11. doi:10.1186/s12890-016-0242-0

21 University of Texas School of Public Health. Search filters for case-control studies, cohort studies, cross-sectional studies, clinical trials. 2012.http://libguides.sph.uth.tmc.edu/search_filters/ovid_psycinfo_filters (accessed 13 Jun 2017).

22 Scottish Intercollegiate Guidelines Network. SIGN search filters for observational studies.

23 BMJ Evidence Centre. Medline cohort and case-control strategy. 2012.http://clinicalevidence.bmj.com/x/set/static/ebm/learn/665076.html (accessed 13 Jun 2017).

24 Moher D, Liberati A, Tetzlaff J, *et al.* Preferred Reporting Items for Systematic Reviews and Meta-Analyses: The PRISMA Statement. *Annu Intern Med* 2009;**151**:264–9. doi:10.1371/journal.pmed1000097

25 World Health Organization. International Clinical Trials Registry Platform Search Portal. http://apps.who.int/trialsearch/ (accessed 13 Jun 2017).

26 Kirkham JJ, Dwan KM, Altman DG, *et al.* The impact of outcome reporting bias in randomised controlled trials on a cohort of systematic reviews. *BMJ* 2010;**340**:c365. doi:10.1136/bmj.c365

27 Guyatt GH, Oxman AD, Vist GE, *et al.* GRADE: an emerging consensus on rating quality of evidence and strength of recommendations. *BMJ* 2008;**336**:924–6. doi:10.1136/bmj.39489.470347.AD

For peer review only

PRISMA-P (Preferred Reporting Items for Systematic review and Meta-Analysis Protocols) 2015 checklist: recommended items to address in a systematic review protocol*

Section and topic	Item No	Checklist item	Status	Page
**ADMINISTRATIVE INFORMATION**				
Title:				
Identification	1a	Identify the report as a protocol of a systematic review	Completed	1
Update	1b	If the protocol is for an update of a previous systematic review, identify as such	n/a	n/a
Registration	2	If registered, provide the name of the registry (such as PROSPERO) and registration number	Completed	2, 21
Authors:				
Contact	3a	Provide name, institutional affiliation, e-mail address of all protocol authors; provide physical mailing address of corresponding author	Completed	1
Contributions	3b	Describe contributions of protocol authors and identify the guarantor of the review	Completed	21
Amendments	4	If the protocol represents an amendment of a previously completed or published protocol, identify as such and list changes; otherwise, state plan for documenting important protocol amendments	Completed	17
Support:				
Sources	5a	Indicate sources of financial or other support for the review	Completed	21
Sponsor	5b	Provide name for the review funder and/or sponsor	Completed	21
Role of sponsor or funder	5c	Describe roles of funder(s), sponsor(s), and/or institution(s), if any, in developing the protocol	Completed	21
**INTRODUCTION**				
Rationale	6	Describe the rationale for the review in the	Completed	4-6

For peer review only

		context of what is already known		
Objectives	7	Provide an explicit statement of the question(s) the review will address with reference to participants, interventions, comparators, and outcomes (PICO)	Completed	7
**METHODS**				
Eligibility criteria	8	Specify the study characteristics (such as PICO, study design, setting, time frame) and report characteristics (such as years considered, language, publication status) to be used as criteria for eligibility for the review	Completed	7-9
Information sources	9	Describe all intended information sources (such as electronic databases, contact with study authors, trial registers or other grey literature sources) with planned dates of coverage	Completed	9
Search strategy	10	Present draft of search strategy to be used for at least one electronic database, including planned limits, such that it could be repeated	Completed	9-12
Study records:				
Data management	11a	Describe the mechanism(s) that will be used to manage records and data throughout the review	Completed	13
Selection process	11b	State the process that will be used for selecting studies (such as two independent reviewers) through each phase of the review (that is, screening, eligibility and inclusion in meta-analysis)	Completed	13
Data collection process	11c	Describe planned method of extracting data from reports (such as piloting forms, done independently, in duplicate), any processes for obtaining and confirming data from investigators	Completed	13-14
Data items	12	List and define all variables for which data will be sought (such as PICO items, funding sources), any pre-planned data assumptions and simplifications	Completed	14-15
Outcomes and	13	List and define all outcomes for which data will	Completed	15-16

For peer review only

prioritization		be sought, including prioritization of main and additional outcomes, with rationale		
Risk of bias in individual studies	14	Describe anticipated methods for assessing risk of bias of individual studies, including whether this will be done at the outcome or study level, or both; state how this information will be used in data synthesis	Completed	16-17
Data synthesis	15a	Describe criteria under which study data will be quantitatively synthesised	Completed	17
	15b	If data are appropriate for quantitative synthesis, describe planned summary measures, methods of handling data and methods of combining data from studies, including any planned exploration of consistency (such as I^2, Kendall's τ)	Completed	17-19
	15c	Describe any proposed additional analyses (such as sensitivity or subgroup analyses, meta-regression)	Completed	19
	15d	If quantitative synthesis is not appropriate, describe the type of summary planned	Completed	19-20
Meta-bias(es)	16	Specify any planned assessment of meta-bias(es) (such as publication bias across studies, selective reporting within studies)	Completed	20
Confidence in cumulative evidence	17	Describe how the strength of the body of evidence will be assessed (such as GRADE)	Completed	20

*** It is strongly recommended that this checklist be read in conjunction with the PRISMA-P Explanation and Elaboration (cite when available) for important clarification on the items. Amendments to a review protocol should be tracked and dated. The copyright for PRISMA-P (including checklist) is held by the PRISMA-P Group and is distributed under a Creative Commons Attribution Licence 4.0.**

From: Shamseer L, Moher D, Clarke M, Ghersi D, Liberati A, Petticrew M, Shekelle P, Stewart L, PRISMA-P Group. Preferred reporting items for systematic review and meta-analysis protocols (PRISMA-P) 2015: elaboration and explanation. BMJ. 2015 Jan 2;349(jan02 1):g7647.

For peer review only
